# Amphibian reproductive technologies: approaches and welfare considerations

**DOI:** 10.1093/conphys/coab011

**Published:** 2021-03-16

**Authors:** Aimee J Silla, Natalie E Calatayud, Vance L Trudeau

**Affiliations:** School of Earth, Atmospheric and Life Sciences, University of Wollongong, Northfields Ave, Wollongong, New South Wales 2522, Australia; Taronga Institute of Science and Learning, Taronga Conservation Society Australia, Taronga, Western Plains Zoo, Obley Rd, Dubbo, New South Wales 2830, Australia; San Diego Zoo Global-Beckman Center for Conservation Research, San Pasqual Valley Rd, Escondido, CA 92027, USA; Department of Biology, University of Ottawa, Ottawa, Ontario K1N 6N5, Canada

**Keywords:** Assisted reproductive technologies, captive breeding, endocrinology, genetic management, reproduction, spawning

## Abstract

Captive breeding and reintroduction programs have been established for several threatened amphibian species globally, but with varied success. This reflects our relatively poor understanding of the hormonal control of amphibian reproduction and the stimuli required to initiate and complete reproductive events. While the amphibian hypothalamo–pituitary–gonadal (HPG) axis shares fundamental similarities with both teleosts and tetrapods, there are more species differences than previously assumed. As a result, many amphibian captive breeding programs fail to reliably initiate breeding behaviour, achieve high rates of fertilization or generate large numbers of healthy, genetically diverse offspring. Reproductive technologies have the potential to overcome these challenges but should be used in concert with traditional methods that manipulate environmental conditions (including temperature, nutrition and social environment). Species-dependent methods for handling, restraint and hormone administration (including route and frequency) are discussed to ensure optimal welfare of captive breeding stock. We summarize advances in hormone therapies and discuss two case studies that illustrate some of the challenges and successes with amphibian reproductive technologies: the mountain yellow-legged frog (*Rana muscosa*; USA) and the northern corroboree frog (*Pseudophryne pengilleyi*; Australia). Further research is required to develop hormone therapies for a greater number of species to boost global conservation efforts.

## Teaser

Many amphibian captive breeding programs exhibit reproductive failure; however, an increasing number of species are being propagated and managed effectively with the aid of reproductive technologies. Here we review aspects of the amphibian hypothalamo–pituitary–gonadal axis, discuss advances in hormone therapies and draw attention to animal welfare considerations.

## Introduction

Unprecedented rates of species decline have resulted in the sixth mass extinction currently threatening global biodiversity and ecosystem functioning. All vertebrate classes have been affected, though amphibians are exhibiting the most dramatic declines, with an estimated 41% of species threatened with extinction (IUCN, 2020). A moral and ethical obligation now exists to assist threatened species recovery and protect species from further decline (Minteer and Collins, 2013). In response to the amphibian biodiversity crisis, several important interventionist conservation actions have been recommended, including the establishment of *ex situ* captive breeding and reintroduction programs (Gascon et al., 2007). Captive breeding and reintroduction programs (also known as conservation breeding programs) have been established for several threatened and endangered amphibian species globally, assisting to prevent species extinction by maintaining genetically representative captive assurance colonies, while providing individuals for release (Silla and Byrne, 2019). A number of programs report partial to high success rates following the provision of traditional methods of environmental manipulation to induce reproduction in captivity. For other species, replicating the specific environmental stimuli required for reproduction has been unsuccessful, and these programs have been hindered by an inability to reliably and predictably initiate amphibian breeding behaviour, achieve high rates of fertilization or generate large numbers of genetically diverse and viable offspring (Kouba *et al*., 2009; Silla and Byrne, 2019). Reproductive technologies are increasingly being adopted to enhance the propagation and genetic management of amphibian conservation breeding programs (Clulow *et al*., 2014, 2019; Browne *et al*., 2019; Silla and Byrne, 2019; Silla *et al*., 2018, 2019). They encompass a range of techniques such as hormone therapies, gamete storage, biobanking and *in vitro* fertilization (IVF), which can be used as tools to enhance reproductive success and promote genetic potential within managed populations (Silla and Byrne, 2019). While reproductive technologies can be used to control the reproductive physiology of individuals in captivity, we advocate a more holistic approach, discussing the use of these technologies to complement rather than substitute traditional captive breeding methods. Providing naturalistic environmental conditions (including climatic cycling, adequate nutrition and social cues) prior to, or during, the application of reproductive technologies may improve the efficiency of reproductive technologies and, in turn, the sustainability of amphibian conservation breeding programs.

This review begins by introducing the importance of amphibian conservation breeding programs and the role of reproductive technologies. An understanding of reproductive neuroendocrinology is paramount before applying reproductive technologies. Part 1: *Hormonal control of amphibian reproduction*, covers the neuroendocrine system of amphibians, focusing on the proximate mechanisms known to control ovulation and sperm release. Additionally, this section discusses the ways in which stress hormones (corticosteroids) potentially interfere with reproductive hormones. Part 2: *The provision of environmental conditions to enhance reproductive technologies*, does not aim to present exhaustive information on species-specific environmental requirements, but instead focuses on the main environmental factors to consider to complement the application of reproductive technologies (climatic cycling, nutrition and opportunities for mate-choice). Aspects of reproductive cyclity on specific species are beyond the scope of this review. The reader is directed towards previous reviews (Eisthen and Krause, 2012; Vu and Trudeau, 2016; Di Fiore *et al*., 2020) that have covered the topic. Part 3: *Amphibian reproductive technologies*, focuses on the practical application of reproductive technologies. Welfare considerations are discussed including animal handling and restraint, routes of hormone administration, the frequency of hormone injections and the frequency with which reproduction can be induced annually. Animal welfare considerations are often omitted from reviews on amphibian reproductive technologies, however, animal welfare should always be considered of utmost importance. Additionally, if these factors are not carefully considered, hormone therapies may not be effective and the long-term viability of conservation breeding programs will likely be compromised. This section concludes by summarizing current advances in hormone therapies. Finally, Part 4: *Case studies*, presents case studies for the successful incorporation of reproductive technologies into the conservation breeding programs of two threatened amphibians: the mountain yellow-legged frog (*Rana muscosa*) and the northern corroboree frog (*Pseudophryne pengilleyi*).

### Amphibian declines and conservation breeding programs

There are currently more than 8, 000 described amphibian species (IUCN, 2020), of which ~88% are anurans (frogs and toads), 9% urodeles (salamanders and newts) and 3% gymnophions (caecilians). The number of newly described species increases annually, so does the number of species exhibiting population declines and disappearances. Land use changes, invasive species, overexploitation, pollution, climate change and emerging infectious diseases have been identified as the main drivers of amphibian decline and extinction (Hussain, 2012). The rapid emergence of the highly virulent amphibian chytridiomycosis has been particularly devastating, though, despite global research efforts, mitigating the effects of this pathogen remains a major challenge (Berger *et al*., 2016). Concerns over the long time frame required to ameliorate *in situ* processes has led to the recommendation that *ex situ* captive breeding colonies be established for all species at imminent risk of extinction [see the Amphibian Conservation Action Plans (Gascon *et al*., 2007; Wren *et al*., 2015)].

Amphibian conservation breeding programs serve to maintain genetically representative colonies of threatened species outside of their natural habitat as an insurance against imminent extinction, with the long-term objective of providing offspring for reintroductions *in situ* once threatening processes have been abated (McFadden *et al*., 2018; Pritchard *et al*., 2012). Conservation breeding programs have been established for a variety of amphibians globally; however, despite some celebrated achievements, the total number of programs and their success remains insufficient to combat the scale of global amphibian declines (Silla and Byrne, 2019). Fundamental to program success is the ability to efficiently and predictably initiate breeding in captivity and generate large numbers of viable, genetically diverse offspring for release. Reproductive failures in captive amphibians are common as success is dependent on a complex combination of environmental cues (including an array of abiotic and biotic stimuli) that activate the HPG axis (Vu and Trudeau, 2016; Silla and Byrne, 2019). To exacerbate the issue, environmental requirements are highly species-specific and for many threatened species, natural reproductive ecologies and the specific environmental cues required for reproduction, are largely unknown.

### The decision to adopt reproductive technologies

There are three main reasons to incorporate reproductive technologies into amphibian captive breeding and reintroduction programs: (i) to enhance the propagation and genetic management of species exhibiting reproductive failure in captivity, (ii) to improve the genetic management of species that already breed successfully in captivity (through environmental stimulation) and (iii) to improve the reproductive health of individuals. First, the administration of exogenous hormones (hormone therapies) can help to overcome the inhibition of natural breeding, improve spawning rates and/or fertilization success and coordinate spawning of captive populations (Kouba *et al*., 2009; Silla and Byrne, 2019). For species that do not breed reliably in captivity, using reproductive technologies may provide a tool to achieve the propagation and release of individuals into the wild and improve the success of conservation breeding programs (Silla and Byrne, 2019; Bronson and Vance, 2019).

Alternatively, for several amphibian species (including both common and threatened species), applying appropriate environmental conditions conducive to reproduction has been effective at stimulating successful captive breeding without the need for further intervention using hormone injections. One example is the Mallorcan midwife toad (*Alytes muletensis*), which is the subject of a highly successful captive breeding program coordinated by the Durrell Wildlife Conservation Trust, Jersey, UK. The program has led to the generation of 18 self-sustaining wild populations and doubling of the species’ geographical range, resulting in the downgrading of the species from ‘critically endangered’ to ‘vulnerable’ in the IUCN Red List (Griffiths *et al*., 2008). Despite these achievements, concerns have been raised over the deterioration of fitness-determining traits and genetic variation in long-term captive colonies of this species (Kraaijeveld-Smit *et al*., 2006). The loss of genetic diversity and adaptive capacity over time is a common problem among captive populations of many species (Taylor, 2003). Reproductive technologies have the potential to aid the genetic management of threatened species in captivity in a variety of ways. The generation of offspring from genetically valuable parents can be promoted either by selecting male–female pairs and inducing spawning through hormonal induction (Silla *et al*., 2018), or using IVF technologies (Silla and Byrne, 2019). IVF requires collecting freshly ovulated oocytes from females following hormone therapy and fertilizing them using either freshly collected hormone-induced sperm, or thawed cryopreserved sperm (Silla and Byrne, 2019). IVF also allows the application of split-clutch factorial mating designs, which enable a group of males and females to be crossed in every pairwise combination (e.g. Byrne *et al*., 2019; Byrne and Silla, 2020), generating offspring with maximum genetic diversity (Silla and Byrne, 2019).

Reproductive technologies may also be adopted in order to maintain the health and wellbeing of captive amphibians. For example, in females, egg retention (also known as becoming egg bound) is a common reproductive disorder resulting from an inability to oviposit some or all of the eggs (Whitaker, 2001; Gupta *et al*., 2015). Egg retention can occur due to the presentation of an incompatible or undesirable mating partner, insufficient environmental stimuli to promote oviposition, an episode of acute stress before or during oviposition or the onset of illness (Whitaker, 2001). Hormone therapy may be used to ensure the complete deposition of eggs (Bronson and Vance, 2019; Calatayud *et al*., 2019; Kouba *et al*., 2012b) and avoid health issues such as dehydration, bacterial infection, the development of ovarian cysts or septicemia, which may result in female mortality (Fitzgerald *et al*., 2007; Whitaker, 2001).

## Part 1. Hormonal control of amphibian reproduction

**Figure 1:**
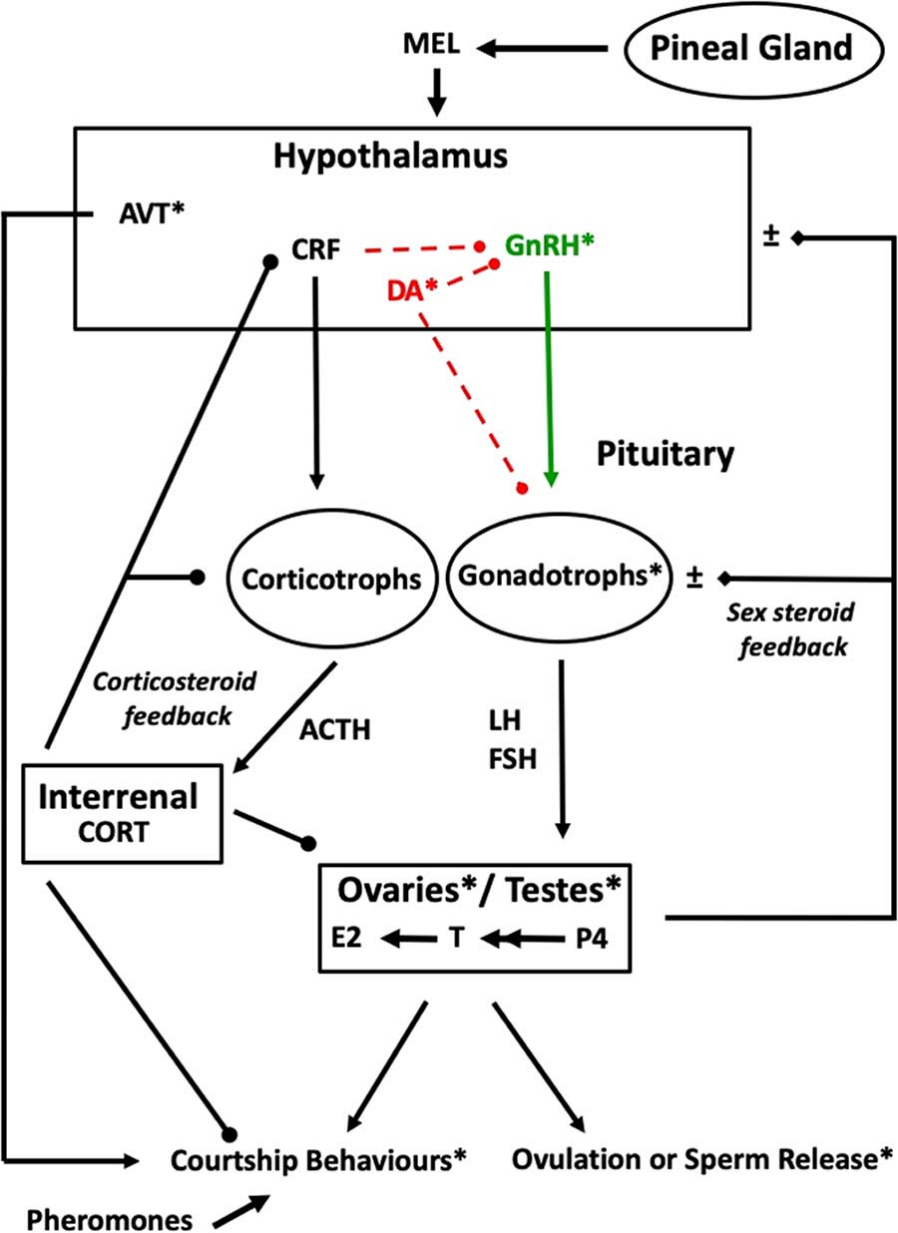
Proposed simplified model for the neuroendocrine control of spawning in amphibians. The principal stimulatory neuropeptide gonadotropin-releasing hormone (GnRH) is released from hypothalamic nerve terminals in the median eminence and transported to the anterior pituitary, where it acts on G-protein coupled GnRH receptors on gonadotrophs to synthesize the gonadotropins luteinizing hormone (LH) and follicle-stimulating hormone (FSH). The effects of GnRH are modulated by numerous neurohormones with emerging functions (Vu and Trudeau, 2016). The inhibitory catecholamine dopamine (DA), which may reduce GnRH action and LH release, is shown. Once secreted, circulating LH and FSH act on their respective G-protein coupled receptors in the ovaries or testes to drive steroidogenesis and gamete release. Here, progesterone (P_4_) is converted through multiple enzymatic steps to testosterone (T), which is aromatized to estradiol (E_2_). Estradiol plays an additional role in stimulating the hepatic synthesis of the egg yolk vitellogenin in females (not shown). Sex steroids are involved in gonadal development, reproductive behaviours and both positive and negative feedback control of the hypothalamo–pituitary axis. Also shown are key aspects of the stress axis. The neuropeptide corticotropin-releasing factor (CRF) is released from hypothalamic nerve terminals in the median eminence and transported to the pituitary, where it acts on G-protein coupled CRF receptors on corticotrophs to drive the synthesis and processing of the pre-pro-opiomelaocortin precursor protein to generate the peptide adrenocorticotropic hormone (ACTH). The ACTH is released into the circulation and binds to melanocortin receptors on steroidogenic cells in the interrenal to stimulate corticosterone (CORT) synthesis, which in turn regulates metabolism and immune function and critically exerts negative feedback control on CRF and ACTH cells. Accumulating evidence indicates that CORT can negatively modulate reproductive processes at multiple sites (see * in Figure 1) along the hypothalamo–pituitary–gonadal (HPG) axis, including GnRH and DA neurons, gonadotrophs and gonads. Moreover, hypothalamic CRF neurons may project to GnRH neurons where they could inhibit GnRH release in several vertebrates, but this remains to be determined in amphibians. The neuropeptide arginine vasotocin (AVT) is produced in preoptic magnocellular neurons, can be released to the blood from the posterior pituitary and has critical roles in stimulating reproductive behaviours. The AVT system is a target of steroid action to modulate sexual behaviour. Although not well understood, the pineal hormone melatonin (MEL) is released in a time- and season-dependent manner at night and has a role to play in regulating reproductive cycles. Sex pheromones are not well described in anurans, but in urodeles, at least some are peptides. Arrowheads indicate stimulation, circles indicate inhibition and diamond heads indicate modulatory effects (stimulatory and inhibitory).

The neuroendocrine control of ovulation and sperm release in amphibians is poorly understood and is largely limited to anurans (Vu and Trudeau, 2016), Nevertheless, many of the basic features of the HPG axis are similar across taxa and will be considered here (Fig. 1), as this knowledge is fundamental for careful application and development of hormone treatments to control reproduction in captive amphibians.

### The hypothalamo–pituitary complex

The hypothalamo–pituitary complex in amphibians shares structural and functional similarities with fish, reptiles, birds and mammals (Ball, 1981; Trudeau and Somoza, 2020). The hypothalamus, chiefly the preoptic area, located in the ventral diencephalon is the central regulator of reproduction. Attached to the hypothalamus through an infundibular stalk is the pituitary gland that consists of the neurohypophysis (posterior pituitary) and adenohypophysis (anterior pituitary). The amphibian neurohypophyseal peptides arginine vasotocin (AVT) and mesotocin are respectively the homologs of mammalian vasopressin and oxytocin that are released into systemic circulation. In amphibians, the cells in the anterior pituitary are controlled by hypothalamic neuropeptides and catecholamines released into and transported by the median eminence portal blood system. It is specifically the cells of the pars distalis of the anterior pituitary that biosynthesize and secrete hormones that are involved in growth, reproduction and the endocrine stress response among many other functions (Ball, 1981; Moore, 1987). The pituitary hormones that are directly involved in the production of eggs and sperm, gonadal sex steroid synthesis and spawning are called the gonadotropins. They are luteinizing hormone (LH) and follicle-stimulating hormone (FSH).

### *Amphibian LH and FSH are stimulated by the hypothalamic neuropeptide gonadotropin* hormone-releasing hormone

Both LH and FSH are heterodimeric glycoproteins consisting of a common alpha subunit and a unique beta subunit that confers specificity of hormone action as in other tetrapods. These gonadotropins are biosynthesized by gonadotrophs and are stored in secretory vesicles either separately or together in these cells located in the pars distalis of the anterior pituitary (Gracia-Navarro and Licht, 1987). Both LH and FSH receptors have been partially characterized in bullfrog (*Lithobates catesbeianus*) testes (Yamanouchi and Ishii, 1990) and liver (Kubokawa and Ishii, 1987), in addition to Japanese fire belly newt (*Cynops pyrrhogaster*) testes (Kubokawa and Ishii, 1980). Research progress on the roles of LH and FSH has been hampered by the very limited availability of purified amphibian gonadotropins. Nevertheless, the gonadal steroidogenic functions of LH and FSH are reasonably conserved across the tetrapod classes (Clulow *et al*., 2014; Polzonetti-Magni *et al*., 1998). Final oocyte maturation and testicular androgen production in frogs is regulated by LH, whereas FSH promotes early follicular development. Both LH and FSH have an additional role in female frogs to stimulate the hepatic synthesis and uptake of the egg yolk protein vitellogenin directly and indirectly through increased estradiol-17β (E2) (Licht, 1979; Polzonetti-Magni *et al*., 1998). Across vertebrates, it has been established that an appropriately timed surge of LH is the critical neuroendocrine event to induce ovulation and sperm release (Vu and Trudeau, 2016; Clulow *et al*., 2018).

More than 20 natural gonadotropin-releasing hormone (GnRH) subforms have been identified across vertebrates with diverse functions ranging beyond its primary role to stimulate gonadotropin release. The general structure of the natural mammalian decapeptide is characterized as pGlu-His-Trp-Ser-Tyr-Gly-Leu-Arg-Pro-Gly-NH_2_, where variants are distinguished by amino acid substitutions (Millar *et al*., 2004). Phylogenetic analyses have classified the multitude of GnRH subforms into three main branches: GnRH-I, GnRH-II and GnRH-III (Millar *et al*., 2004), historically known as mammalian GnRH (mGnRH), chicken GnRH-II (cGnRH-II) and salmon GnRH (sGnRH) forms. High-performance liquid chromatography and immunological analysis revealed that there are typically two of these GnRH variants in many species (Vu and Trudeau, 2016). The two most common forms that occur in amphibian brains are GnRH-I and GnRH-II (Licht *et al*., 1994). Studies in *Lithobates pipiens*, *Pelophylax ridibundus* and *Pelophylax esculentus* have revealed that GnRH-II widely distributed in the brain and spinal cord and is therefore suspected to be involved in neuromodulatory control and sexual behaviours; however, the expression of GnRH-I is restricted to the anterior preoptic area of the hypothalamus and the median eminence, suggesting that GnRH-I is the primary hypophysiotropic form stimulating the synthesis and release of gonadotropins (Licht *et al*., 1994), and thus controlling spawning.

The neuropeptide GnRH is released from axon terminals in the amphibian median eminence, transported by portal blood vessels to the pars distalis where they exert their stimulatory actions through membrane-bound G-coupled protein GnRH receptors (GnRHRs) found on gonadotrophs. Three distinct GnRHRs have been identified in the bullfrog that exhibit differential ligand selectivity and intracellular signalling (Wang *et al*., 2001, 2003). GnRHR-1 is the prominent subtype in the pituitary whereas GnRHR-2 and -3 mRNAs are expressed in the brain, suggesting additional roles, apart from the control of reproductive behaviour (Wang *et al*., 2001). The GnRHR-1 has the highest affinity for GnRH-II, followed by GnRH-III and GnRH-I (Wang *et al*., 2001). Understanding and taking advantage of these key aspects of GnRH physiology will be essential to improve current technologies and reproductive outcomes and contribute to the welfare of captive species. This would be achieved by optimization of treatment protocols with minimal handling and intervention.

### Dopamine as an inhibitory regulator of reproduction

Emerging evidence points to an inhibitory role of the catecholamine dopamine (DA) in the control of spawning in amphibians (reviewed in Vu and Trudeau, 2016). In teleost fish, DA acts to inhibit the release of gonadotropins from the pituitary and to attenuate the stimulatory actions of GnRH (Dufour *et al*., 2005). This mechanism is suspected to be evolutionarily conserved and active in some amphibians. A series of studies conducted in hibernating grass frogs (*Rana temporaria*) have confirmed the existence of inhibitory hypothalamic control where LH release and ovulation were induced following electrolytic lesions in the nucleus infundibularis ventralis (Sotowska-Brochocka, 1988; Sotowska-Brochocka and Licht, 1992). The administration of the DA antagonist metoclopramide (MET) in hibernating grass frogs triggered ovulation, whereas the DA agonist bromocriptine reduced the release of LH (Sotowska-Brochocka *et al*., 1994). Creighton et al. (2014) have suggested that DA is involved in male motor behaviours and vocalization. Male green tree frogs (*Hyla cinerea*) displayed decreased calling rates and were less likely to engage in activities such as climbing following injections of the specific dopamine receptor D2 agonist quinpirole (Creighton and Wilczynski, 2014). On the other hand, quinpirole treatments delay or inhibit oviposition and spawning in wood frogs (*Lithobates sylvaticus*) (Vu and Trudeau, 2016)*.* The combined single injection of a GnRH agonist (GnRH-a) and a D2 antagonist results in maximal spawning rates in leopard frogs (*L. pipiens*) (the AMPHIPLEX method; Trudeau *et al*., 2010, 2013) and has been effective in a number of other amphibians (Clulow *et al*., 2014; Clulow *et al*., 2018, Vu and Trudeau, 2016). Treatment with the D2 agonist metoclopramide enhances the stimulatory effect of GnRH-a to increase mRNA levels of LHβ and FSHβ gonadotropin subunits and the type 1 GnRH receptor in the female leopard frog pituitary gland (Vu *et al*., 2017). While definitive evidence requires more experimentation, these data support the proposal that DA inhibits gonadotropin synthesis and secretion in anurans, as it does in teleosts (Dufour *et al*., 2005, Popesku *et al*., 2008). Together, these data suggest that DA is involved in inhibiting spawning and mate advertisement in some amphibians. Factors in captive environments that potentially increase inhibitory dopaminergic tone may contribute to reduced reproductive outputs, but this speculation requires rigorous testing. In the case of spawning induction in teleosts the inclusion of DA antagonists with GnRH agonists remains the most effective, injectable method for reproductive enhancement for a multitude of species (Dufour *et al*., 2010; Peter *et al*., 1988).

### Role of sex steroids in the control of amphibian reproduction

Sex steroids are essential for sexual behaviours, gonadal maturation and the development of secondary sexual characteristics in adults. Steroidogenesis is controlled largely by the actions of LH and FSH on the testes or ovaries. In female amphibians, three classes of sex steroids—the estrogens, progesterone and androgens—are primarily synthesized by ovarian follicles that are composed of thecal and granulosa cell layers. Granulosa cells synthesize progesterone, 17α-hydroxyprogesterone (17α-OHP_4_) and androstenedione (A_4_) that is subsequently converted to testosterone (T) in the thecal cells. Granulosa cells have an additional role in converting T to estradiol (E_2_) through the actions of aromatase. The main steroidogenic sites in amphibian testes are the interstitial Leydig cells and Sertoli cells, in addition to lobule boundary cells that occur exclusively in urodeles. Predominantly, it is the androgens T and 5α-dihydrotestosterone (5α-DHT) that are synthesized and secreted by the Leydig cells. Testosterone is aromatized to E_2_ in the Sertoli cells (Ogielska, 2009). Both negative and positive sex steroid feedback effects have been reported *in vivo* and *in vitro* for several amphibian species (Vu and Trudeau, 2016). Both androgens and estrogens exert their major actions through the nuclear androgen and estrogen receptors, which are liganded transcription factors expressed on neurons in the hypothalamus and gonadotrophs in the pituitary. These classical steroidal feedback actions are illustrated in Fig. 1.

There is a clear positive relationship between testicular androgens and male sexual activity, a generalization that holds true for both anurans and urodeles. Comparable studies involving castration followed by replacement with T or 5α-DHT have demonstrated that amplectic clasping and advertisement calls are highly dependent on circulating androgen levels (Vu and Trudeau, 2016). Reproductive behaviours in females are similarly controlled by sex steroids. One of the most common behaviours exhibited by female amphibians is phonotaxis. In response to male advertisement calls, females will move towards these signals and strong evidence suggests that this receptive behaviour is mediated primarily by E_2_. Currently, there is greater emphasis on the study of male amphibian courtship behaviours; however, the neuroendocrine regulation of female behaviours is an avenue that remains to be further explored (Vu and Trudeau, 2016), especially within the context of captive breeding.

### Corticosterone is an interrenal corticosteroid that can negatively regulate reproduction

Corticosteroids may be important for the regulation of sexual behaviour and reproduction (Chand and Lovejoy, 2011). Corticotropin-releasing factor (CRF) is secreted from the hypothalamus to trigger the release of adrenocorticotropic hormone (ACTH) from the anterior pituitary and corticosterone (CORT) from the steroidogenic cells of the interrenal (amphibian equivalent to mammalian adrenal cortex embedded within the kidney complex). The hypothalamo–pituitary–interrenal (HPI) axis is a classical neuroendocrine stress axis, where corticosteroids, in turn, negatively feedback at the level of CRF and ACTH to attenuate glucocorticoid synthesis. Corticosterone is the main adrenal steroid in amphibians, reptiles, birds and rodents, whereas cortisol dominates in teleosts and other mammals (Cockrem, 2013).

Increases in plasma CORT during breeding have been documented for several amphibians (Romero, 2002, Moore and Jessop, 2003), including the Argentinian toad (*Rhinella arenarum*; Denari and Ceballos, 2005) and several Brazilian desert toads (Madelaire and Gomes, 2016), among others. Urinary steroids show similar associations in the Bombay night frog (*Nyctibatrachus humayuni*; Joshi *et al*., 2018). This relationship is less clear in some other species (de Assis *et al*., 2012; Houck *et al*., 1996). Corticosteroid administration under experimental conditions and/or the natural rise in CORT are often inversely correlated with androgen levels in frogs and salamanders (Licht *et al*., 1983, Woodley and Lacy, 2010). In the newts *Taricha granulosa* and *Notophthalmus viridescens*, CORT has been shown to inhibit amplexus within a few minutes (Davis *et al*., 2015; Moore and Miller, 1984).

Plasma CORT concentrations in wild female whistling frogs (*Litoria ewingii*) and bullfrogs increase with capture time (Coddington and Cree, 1995; Licht *et al*., 1983). Moreover, corresponding gonadotropin and sex steroid levels are highly sensitive to capture and handling stress. In *T. granulosa, L. catesbeianus* and *Bufo marinus,* confinement has been consistently accompanied by a suppression of circulating androgens and gonadotropins (Licht *et al*., 1983; Mendonça *et al*., 1985; Moore and Zoeller, 1985; Orchinik *et al*., 1988). On the other hand, supraphysiological concentrations of CORT had no effect on sperm count or viability in male White’s tree frog (*Litoria caerulea*; Kaiser *et al*., 2015).

There are multiple sites of action (Fig. 1) by which the stress axis can influence the reproductive axis in vertebrates (Chand and Lovejoy, 2011). Physiologically relevant breeding season levels of CORT reduce *in vitro* testicular androgen production in *R. arenarum* (Czuchlej *et al*., 2019), providing the only evidence thus far in amphibians of the direct influence of the stress hormone on gonadal function. Nuclear glucocorticoid receptors (GRs) are localized to regions of the hypothalamic preoptic area and pituitary pars distalis of *Xenopus laevis*, but the identity of these target cells remains to be defined (Yao *et al*., 2008). In rainbow trout (*Oncorhynchus mykiss*), however, both GR and estrogen receptor alpha (ERα) were found to be co-expressed in the dopaminergic neurons inhibiting LH secretion and also in pituitary gonadotrophs (Teitsma *et al*., 1998), providing strong anatomical evidence for stress-reproduction interactions. Preoptic DA neurons likely mediate the inhibitory effects of stress on LH release in tilapias (*Oreochromis mossambicus*; Chabbi and Ganesh, 2015). It is tempting to speculate that similar mechanisms are at work in amphibians, but this requires rigorous testing. Most data support the hypothesis that high CORT has a negative effect on reproductive processes in amphibians. It is therefore important that potential stress as indicated by elevated CORT be managed (see Romero *et al*., 2009 and the reactive scope model) to avoid detrimental effects on general health, and particularly on reproduction, for amphibians in captive breeding programs. There is clearly a research necessity to examine interactions between the HPI and HPG axes in Amphibia. This should not only involve development of novel CORT assay methods (including non-invasive assessments), but importantly, the examination of normal roles of corticosteroids in reproduction and under stressful or disease-related conditions. Shown in Fig. 1 are some of the possible sites of action of corticosteroids in the HPG axis, emphasizing the need to optimize the environment provided to amphibians in captivity to minimize negative stress effects on reproduction.

## Part 2: The provision of environmental conditions to enhance reproductive technologies

In order to stimulate the cascade of hormonal changes required to initiate reproductive events, captive facilities aim to identify and recapitulate key environmental elements for reproduction. This is not a trivial undertaking, because amphibians display extreme reproductive diversity, with anurans and urodeles exhibiting 39 and 10 different reproductive modes, respectively (Wells, 2007). Reproductive mode refers to a combination of traits including oviposition site, egg and clutch characteristics, rate and duration of development, stage and size at hatching and presence and type of parental care (Haddad and Prado, 2005). Amphibians, particularly the anurans, exhibit a remarkable level of reproductive diversity (reflected by the number of distinct reproductive modes) that is unique among tetrapod vertebrates (Haddad and Prado, 2005). The environmental cues required to optimize reproductive outcomes are therefore often species-specific. Replicating the complex combination of environmental cues and conditions required to generate viable offspring for each target species can be very difficult. This challenge is exacerbated due to a lack of knowledge of the natural reproductive ecologies of many threatened amphibians, in addition to both financial and space constraints within captive facilities. The species-specific requirements and benefits of various environmental factors require greater research attention and is important for both natural reproduction and the use of reproductive technologies. Reproductive technologies have the potential to bypass some impediments to natural mating and fertilization that animals in captivity often encounter (Silla and Byrne, 2019). However, it is important to note that hormone therapies are likely to be most successful when used in concert with traditional captive breeding methods (e.g. manipulating environmental conditions) that have been refined for the target species. This section briefly describes the main environmental factors to consider to complement reproductive technologies.

### Cycling climatic conditions

Amphibian reproduction is closely linked with their abiotic environment, particularly temperature, rainfall, humidity and photoperiod. If specific requirements are unknown for a species, replicating/cycling conditions reflective of the location that the species’ naturally inhabits is a good starting point (Pramuk and Gagliardo, 2012; Gupta *et al*., 2015). Temporal patterns of amphibian reproduction can be broadly classified as either continuous (aseasonal) or seasonal. Continuous or year-round breeding is typically observed in tropical environments, though breeding activity still tends to peak and trough with variation in climatic conditions, particularly rainfall (Wells, 2007). Most amphibians are classified as seasonal breeders, ranging from explosive breeders where reproduction is restricted to days, to prolonged seasonal breeders where reproduction can occur over weeks to months when environmental conditions are favourable (Wells, 2007). Seasonal reproduction in amphibians is finely tuned to annual phenological variation, particularly changes in temperature and rainfall. Out of the breeding season (such as during colder months or the dry season), some amphibians aestivate or brumate, strategies that capitalize on the body’s ability to survive under reduced metabolic conditions. Aestivation is a reduction in activity and metabolism in response to high temperatures or dry conditions, typically in arid and semi-arid environments where rainfall is scarce and highly variable (Jorgensen, 1992). Brumation (also referred to as hibernation or overwintering) is exhibited by amphibians inhabiting high latitudes or high altitudes, where metabolic rates decrease in response to prolonged exposure to cold (Wells, 2007). While the terms brumation and hibernation are often used interchangeably to refer to amphibians overwintering, amphibians do not truly hibernate, the correct terminology to describe ectothermic animals entering a state of cold-induced torpor is brumation.

Induced aestivation or brumation are often omitted from amphibian captive husbandry practices due to concerns that it may reduce body condition or increase mortality (Carey *et al*., 2005). However, there is increasing evidence for the importance of cooling captive amphibians that would naturally exhibit brumation in the wild. In seasonal breeders, brumation affects vitellogenesis and egg development in females and spermatogenesis in males (Chieffi *et al*., 1980; Lehman, 1977; Smalley and Nace, 1983). Brumation has been shown to influence fat deposition, sexual maturation and reproductive success in boreal toads (*Anaxyrus boreas boreas*) and mountain yellow-legged frogs (*R. muscosa*) (Roth *et al*., 2010; Santana *et al*., 2015; Calatayud et al., 2015a). Temperature appears to be the primary stimulus inducing these effects, though there is evidence that photoperiod may be an important co-factor and lighting duration should be altered correspondingly (Chieffi *et al*., 1980; Borah *et al*., 2019). While oviposition can be hormonally induced in some seasonal breeding amphibians without prior brumation through the use of priming hormone doses (Kouba *et al*., 2012b) in others species such as the boreal toad, hormone therapy is ineffective in the absence of a brumation period (Calatayud et al., 2015a). Additionally, replicating natural environmental stimuli in captivity may be important for reintroduction success, for example, brumation has been reported to increase juvenile survival of mountain yellow-legged frogs following release (Calatayud *et al*., 2020). As such it is recommended that amphibians that naturally undergo aestivation or brumation should be exposed to representative climatic cycling. To avoid excessive stress and immunosuppression, the duration of aestivation/brumation may be shortened in captivity compared with natural conditions (Calatayud *et al*., 2015a, 2020). Additionally, amphibians may be bathed in a prophylactic antifungal treatment prior to brumation to avoid fungal infections (Lentini, 2007).

Gradual acclimation periods should be used to allow animals to enter, and later arouse, from brumation slowly in order to avoid physiological stress. Most importantly, temperature should be altered gradually (<5°C per day) until the desired temperature is reached (see Calatayud *et al*., 2016; Lentini 2007). Prior to brumation, food availability should be reduced as temperature is decreased, and feeding ceased entirely immediately prior to and during brumation, to ensure that undigested food does not remain in the gut and compromise animal health (Whitaker, 2001). Photoperiod should also be decreased prior to brumation to reflect natural changes. Since there is no evidence that amphibians remain in complete darkness over winter, animals may continue to have exposure to light during the brumation period though artificial lighting should be dimmed, and photoperiod significantly reduced, to reflect natural conditions. The timing and duration of the imposed brumation period should be evaluated on a species-specific basis. Examples of brumation periods in captivity include; Puerto Rican crested toad (*Peltophryne lemur*) for 4–6 weeks (Lentini 2007), southern corroboree frogs (*Pseudophryne corroboree*) for 6–7 weeks (McFadden *et al*., 2013), leopard frogs (*L. pipiens*) for 5–8 weeks (Trudeau *et al*., 2013), boreal toads (*A. boreas boreas*) for 4–24 weeks (Calatayud *et al*., 2015a) and mountain yellow-legged frogs, (*R. muscosa*) for 4–12 weeks (Calatayud *et al*., 2018, 2020).

Amphibians naturally occurring in tropical areas are not exposed to extremes in temperature and photoperiod, but reproduction may be associated with changes in rainfall and humidity (Whittier and Crews, 1987). Rainfall chambers, misting systems and humidifiers can be used to replicate natural changes in these environmental conditions prior to reproduction (see Pramuk and Gagliardo, 2012; Gupta *et al*., 2015).

### Nutrition

It is generally accepted that vertebrate gonadal development and fecundity are affected by essential dietary nutrients. However, the field of amphibian reproductive nutrition lags behind that of other taxa and even basic nutrient requirements for amphibians are largely unknown (Ferrie *et al*., 2014). Diets for captive amphibians currently rely predominantly on live invertebrates that are readily available commercially. Concerns over potential nutrient deficiencies of feeder insects, particularly calcium and vitamins A, D and E has led to the common practice of dusting or gut loading prey with calcium and multivitamin supplements to improve nutritional quality (Ferrie et al., 2014; Pramuk and Gagliardo, 2012).

The nutrient composition of amphibian diets is particularly important for females as they invest heavily in egg production (to offset a lack of parental care in most species), and yolk provisioning is known to influence successful reproduction and survival of offspring (Dziminski *et al*., 2009). Recent research has shown that dietary carotenoid supplementation enhances female fecundity in female red-eyed treefrogs (*Agalychnis callidryas*; Ogilvy *et al*., 2012) and improved reproductive success in pairs of strawberry poison frogs (*Oophaga pumilio*; Dugas *et al*., 2013). In contrast, dietary carotenoids have been shown to shorten time to metamorphosis, but to have no effect on time to sexual maturity or sperm quality in male booroolong frogs (*Litoria booroolongensis*; Keogh et al., 2018a,b). There is an urgent need for further research investigating the nutritional requirements to optimize amphibian captive breeding and the outcomes of reproductive technologies.

In addition to the importance of nutritional quality, the quantity of food provided is essential for conditioning amphibians for reproduction. As mentioned above, food quantity should be gradually reduced prior to brumation or aestivation and ceased completely immediately prior to and during this period to avoid health complications (Whitaker, 2001). Amphibians that do not aestivate or brumate may still experience reduced food availability over cooler or dryer months. Although there may be exceptions to the rule, wild amphibians generally increase prey consumption prior to the breeding season (or in some species immediately after) in order to meet the high energetic demands of gametogenesis, courtship and reproduction. It is therefore recommended that captive amphibians be fed an increased quantity of nutrient-rich food prior to and immediately after the breeding season, but that the provision of food be returned to initial levels shortly after breeding to prevent inappropriate weight gain (McWilliams, 2008). As with the provision of climatic conditions, it is recommended that amount of food supplied to captive individuals should be cycled to mimic the natural conditions a species’ is exposed to in the wild.

### Mate choice

Manipulating the social environment to provide captive individuals with opportunities for mate choice may enhance overall mating success (number of successful matings) and/or reproductive success (fertilization rate, offspring number and viability), and has been suggested as a means of overcoming captive reproductive failure in some taxa (Asa *et al*., 2011). Female mate choice in wild amphibian populations is well documented, with evidence that females prefer males exhibiting phenotypic cues (morphological, acoustic, olfactory or behavioural traits) that indicate direct benefits (e.g. oviposition site, parental care) or indirect genetic benefits (e.g. good or compatible genes) (Byrne and Roberts, 2012). Allowing individuals to actively select partners is consistent with the goals of many modern captive breeding facilities, which aim to promote natural behaviours as a means of enhancing reproductive success, animal well-being and behavioural plasticity (Asa *et al*., 2011). However, allowing mate choice in amphibian captive breeding programs may also compromise genetic goals if individual choices are inconsistent with genetic management objectives (Asa *et al*., 2011). Genetic goals for a captive population are usually specified in terms of the proportion of genetic variation (measured as heterozygosity) to be maintained for a specified time. When provided with the opportunity for mate choice, many amphibians exhibit strong mating biases, with only a small number of preferred males contributing paternity, in addition to a proportion of gravid females failing to reproduce annually if they are not presented with males displaying desirable qualities (Silla *et al*., 2018). As a result, many individuals will fail to contribute to subsequent generations. Additionally, it is often assumed that mate-choice will favour the maintenance of genetic diversity; however, recent genetic studies provide evidence that some amphibians actively select genetically related mates (Cayuela *et al*., 2017; O’Brien *et al*., 2019). Over time, such captive mating preferences and biases may lead to a loss of genetic variation and adaptive potential that could have profound impacts on both captive population viability and reintroduction success. Planned pairings coupled with reproductive technologies, may enhance the efficiency of genetic management and maximize retention of genetic diversity. For example, in the critically endangered northern corroboree frog (*P. pengilleyi*) the mating success (pairs ovipositing) of designated breeding pairs increased from 22% to 100% through the use of hormone injections, resulting in both a greater number of offspring produced and improved genetic representation of the founder population (see case study 2 below; Silla *et al*., 2018). Another strategy that may allow the opportunity for female mate-choice, while still allowing a moderate-to-high level of control over genetic pairings, it to present females with 2–3 potential sires (e.g Trudeau *et al*., 2010). The males presented can be selected based on genetic compatibility or genetic quality to align with genetic management goals.

## PART 3: Amphibian reproductive technologies

### Animal welfare considerations

Once the decision has been made to incorporate reproductive technologies into a captive breeding program, either for propagation, genetic management, or reproductive health purposes, several animal welfare considerations need to be cogitated prior to implementation. Points for consideration include the following: (i) animal handling and restraint, (ii) the route of hormone administration, (iii) the frequency of hormone injections to stimulate reproductive events and (iv) the frequency of induced reproductive events on an annual cycle.

### Animal handling and restraint

When handling and restraining amphibians, the safety of personnel and the well-being of the amphibian must be considered (Wright, 2001). Every effort should be made to minimize stress for the animals during capture and restraint. The skin of many species of anurans and salamanders are known to produce toxic and inflammatory secretions, while in others there are highly adhesive antipredatory mucilaginous secretions (Wright, 2001). Many amphibians, such as dendrobatid frogs, produce toxic compounds generated by metabolizing precursors ingested from their native diet (primarily certain species of ants) and captive-bred individuals are therefore devoid of the toxins (O’Rourke *et al*., 2018). Other species, including some toads and newts, can produce endogenous toxins that in some cases may increase over time in captivity (O’Rourke *et al*., 2018). Hence, talc-free latex or vinyl gloves should be worn whenever an amphibian is handled to minimize contact with defensive secretions. Gloves should also be worn in order to minimize damage and abrasion to the epidermis and eliminate the absorption of toxins such as nicotine or disinfectants that might be on the hands of the handler (O’Rourke *et al*., 2018). Anurans should be restrained by gently grasping immediately anterior to and around the hindlimbs. Salamanders can be secured by grasping both behind the forelimbs and around the hindlimbs, and care should be taken to avoid handling the tail as this can harm the spine and, in rare instances, tail autonomy can occur (Wright, 2001). To access the cloaca during gamete collection (procedures described below), small- to medium-sized anurans and salamanders can be gripped around the mid-section or in a fist. Securing an animal’s anterior body (head and trunk) in a firm but gentle fist has the added advantage of shielding the eyes and restricting vision, which may reduce stress.

Reducing animal stress during handling is another important objective. Recent research on the cane toad (*Rhinella marina*) revealed that manual restraint of individuals for durations of 15 or 30 minutes leads to a significant increase in mean CORT stress response compared to individuals that are unrestrained or restrained for a period of 5 minutes (Narayan *et al*., 2012a). Manual restraint was also shown to result in a significant reduction in urinary T concentrations (Narayan *et al*., 2012a). Restraint should therefore be as short as possible and restricted to periods of less than 5 minutes. The administration of reproductive hormones, either via injection or topical application (see section 4.2.2. below), can be achieved quickly and efficiently in under 2–3 minutes. Restraint time is reduced if technicians are well prepared and hormone syringes are drawn to the correct dose prior to handling the animal. Where breeding pairs of amphibians are hormonally induced to spawn, no further handling should be required. Individuals that are induced to spermiate or ovulate in the absence of a partner will require additional handling and restraint during gamete collection. Sperm collection is achieved post-hormone administration via abdominal massage or cannulation (Browne *et al*., 2019). Urination is a common defensive behaviour exhibited by anurans (Toledo *et al*., 2011) and males of most species will urinate quickly upon restraint allowing sperm to be collected in high concentrations as they are flushed through the cloaca in a mixture of urine and cloacal secretions (spermic urine; Kouba *et al*., 2013; Keogh *et al*., 2015). The collection of oocytes from female amphibians post-hormone administration is facilitated by holding the frog with legs unrestrained and gently applying pressure to the abdomen in a craniocaudal direction (a technique referred to as stripping; Silla 2010). This method replicates natural inguinal and axillary amplexus (where males clasp the female around the waist or armpits, respectively; Wells, 2007). Females that have successfully ovulated in response to hormone administration generally begin to expel oocytes within a couple of minutes of pressure being applied (Byrne and Silla, 2010; Silla, 2011). Where a female amphibian fails to expel oocytes at the expected time, it is recommended that stripping be attempted for no more than 5 minutes, as handling for longer periods is known to lead to a significant increase in stress (Narayan *et al*., 2012a). Stripping can then be reattempted following a rest period of at least 30 minutes. The stress response of cane toads has been shown to significantly decrease on subsequent occasions (at 14-day intervals) (Narayan *et al*., 2012b). Therefore, it is recommended that individuals be well-trained for restraint procedures prior to hormone administration and gamete collection.

### Route of hormone administration

Exogenous hormones are typically administered to anurans via injection intravenously (IV, injection into a vein), intraperitoneally (IP, injection into the peritoneal cavity of the abdomen) or subcutaneously (SC, injection under the skin; SC injections are most commonly administered in the area of the dorsal lymph sac) (Table 1). Intravenous injections, though the most efficient of the three methods (Turner *et al*., 2011), are generally avoided due to the small size of amphibians and their lack of large, readily accessible superficial veins. Small body size also presents a challenge when administering intraperitoneal injections, as care must be taken to avoid inadvertent injection of hormones into the urinary bladder or intestine (Turner *et al*., 2011). Alternatively, SC injections are considered to be less risky (Roth and Obringer, 2003) and easier to master (Turner *et al*., 2011), although appropriate training and supervision is essential to ensure that the personnel performing injections are competent to undertake procedures (NHMRC, 2008). To further promote animal well-being and minimize pain and discomfort, it is recommended that ultrafine (30–31 gauge) needles are employed for injections, irrespective of the route chosen. Single-use sterile syringes should be used and discarded to avoid transmission of disease and infection.

**Table 1:** Examples of successful protocols for the administration of exogenous hormones for gamete release and spawning in amphibians. This table provides an overview of the commonality of hormones used but highlights variability in the routes of administration, hormone doses and number of treatments administered

Species	Sex	Hormone	Route of administration	Effective dose	No. of treatments	Outcome	Reference
*Ambystoma mexicanum *	M, F	hCG	IM injection	200 IU hCG (M) 300 IU hCG (F)	1	Spermiation achieved in 100% (10/10) of males and ovulation achieved in 100% (10/10) of females	(Mansour *et al*., 2011)
*Anaxyrus americanus*	M	hCG	IP injection	300 IU hCG	1	Spermiation achieved in 100% (16/16) of males	(Kouba *et al*., 2012a)
*Anaxyrus americanus*	M	GnRH-a	Topical	100 μg GnRH-a	1	Spermiation achieved in 75% (12/16) of males	(Rowson *et al*., 2001)
*Anaxyrus baxteri*	M	GnRH-a	IP injection	0.2 μg/g GnRH-a	1	Spermiation achieved in 100% (24/24) of males	(Poo *et al*., 2019)
*Anaxyrus boreas boreas*	F	hCG + GnRH-a	IP injection	2x priming 3.7 IU/g hCG + 1x ovulatory 13.5 IU/g hCG + 0.4 μg/g GnRH-a	3	Spawning achieved in 77% (17/22) of hibernated females	(Calatayud *et al*., 2015a)
*Anaxyrus fowleri*	M	hCG	IP injection	300 IU hCG	1 every 1–3 weeks (3–9 in total)	Spermiation achieved in 80–100% of males (*n* = 5–6) after each injection	(McDonough *et al*., 2016)
*Anaxyrus fowleri*	F	GnRH-a + P_4_	IP injection	60 μg GnRH-a + 5 mg P_4_	1	Ovulation achieved in 85% (6/7) of females	(Browne *et al*., 2006)
*Atelopus zeteki*	M	GnRH-a + MET	IP injection	0.4 μg/g GnRH-a + 10 μg/g MET	1	Spermiation achieved in 100% (24/24) of males	(Della Togna *et al*., 2017)
*Crinia glauerti* *Crinia georgiana* *Crinia pseudinsignifera*	M	GnRH-a	SC injection	2 μg/g GnRH-a	1	Spermiation achieved in 100% (*n* = 6–8 per species) of males	(Silla and Roberts, 2012)
*Eleutherodactylus coqui*	F	GnRH-a	SC injection	20 μg GnRH-a	1	Ovulation achieved in 67% (10/15) of females	(Michael *et al*., 2004)
*Geocrinia rosea*	M	GnRH-a	SC injection	1 μg GnRH-a	1	Spermiation achieved in 100% (10/10) of males	(Silla *et al*., 2020)
*Geocrinia rosea*			Topical	100 μg GnRH-a	1	Spermiation achieved in 100% (10/10) of males	(Silla *et al*., 2020)
*Lithobates pipiens*	M,F	GnRH-a + MET	IP injection	0.4 μg/g GnRH-a + 10 μg/g MET	1	Spawning achieved in 89% (16/18) of pairs	(Trudeau *et al*., 2013)
*Lithobates sevosa*	F	GnRH-a + MET	IP injection	0.4 μg/g GnRH-a + 10 μg/g MET	1	Ovulation achieved in 73% (8/11) of females	(Graham *et al*., 2018)
*Lithobates sevosa*		GnRH-a + hCG	IP injection	2x priming 3.7 IU/g hCG + 1x ovulatory 0.4 μg/g GnRHa + 13.5 IU/g hCG	3	Ovulation achieved in 73% (8/11) of females	(Graham *et al*., 2018)
*Litoria booroolongensis*	M	hCG	SC injection	40 IU/g hCG	1	Spermiation achieved in 100% (20/20) of males	(Silla *et al*., 2019)
							
*Pleurodeles waltl*	M	GnRH-a or hCG	IM injection	50 μg GnRH-a or 500 IU hCG	1	Spermiation achieved in 100% (*n* = 3–4) of males	(Uteshev *et al*., 2015)
*Pseudophryne guentheri*	M	GnRH-a	SC injection	2 μg/g GnRH-a	1	Spermiation achieved in 100% (10/10) of males	(Silla, 2010)
*Pseudophryne guentheri*	F	GnRH-a	SC injection	1x priming 0.4 μg/g GnRH-a + 1x ovulatory 2 μg/g GnRH-a	2	Ovulation achieved in 100% (8/8) of females. This protocol has also been successfully used for *P. bibronii*, *P.coriacea* and *Helioporus eyrei*; see Silla and Byrne 2021.	(Silla, 2011)
*Pseudophryne pengilleyi*	M, F	GnRH-a	SC injection	0.5 μg/g GnRH-a	1	Spawning achieved in 100% (9/9) of pairs	(Silla *et al*., 2018)
*Pseudophryne pengilleyi*			Topical	25 μg GnRH-a	1	Spawning achieved in 77% (10/13) of pairs	(Silla *et al*., 2018)
							
*Xenopus laevis*	F	PMSG + hCG	SC injection	1x priming 100 IU PMSG + 1x ovulatory 500 IU hCG	2	Ovulation achieved in 62% (18/29) of females	(Ogawa *et al*., 2011)
*Xenopus laevis*		steroids (P_4_ + E_2_)	Water bath	20 μM P_4_ + 10 μM E_2_	1	Ovulation achieved in 83% (20/24) of females	(Ogawa *et al*., 2011)

Abbreviations: E_2_, estradiol; GnRH-a^b^, gonadotropin-releasing hormone agonist; hCG, human chorionic gonadotropin; IM, intramuscular; IP, intraperitoneal; MET, metoclopramide; PMSG, pregnant mare’s serum; P_4_, progesterone; SC, subcutaneous.

a ^a^Select publications developing hormone therapies for amphibians between 2001 and 2020 are presented.

b ^b^Note that numerous different forms of synthetic GnRH agonists exist with differing biological potencies, so original articles should be consulted for specific details.

A non-invasive alternative to hormone injection is the topical application of exogenous peptide hormones directly to the skin surface (epicutaneous administration). Amphibians possess highly permeable, hypervascularized ventral abdominal surfaces and the application of a GnRH-a directly to the ventral abdomen has been shown to be effective at stimulating spermiation in American and gulf coast toads (*Anaxyrus (Bufo) americanus* and *Incilius (Bufo) valliceps*; Rowson *et al*., 2001) and roseate frogs (*Geocrinia rosea*; Silla *et al*., 2020) and has been successful at inducing spawning in northern corroboree frogs (*P. pengilleyi*; Silla *et al*., 2018). The topical administration of reproductive hormones in combination with dermal penetration enhancers (e.g. acetone, dimethyl sulfoxide) has also been tested, though there is no evidence that these substances improve response rates (Rowson *et al*., 2001) and they may cause skin irritation (Silla *et al*., 2020). In addition to topical application, the addition of sex steroids to a water bath has been successful at inducing ovulation and spawning in the African clawed frog (*X. laevis*; Miyazaki and Tokumoto, 2013; Ogawa *et al*., 2011). However, this protocol is far less cost-effective compared with other methods due to the volume of hormone solution required. Topical and water-bath hormone applications eliminate the need for injection and reduce animal handling, therefore offering a less invasive approach to hormone application that may promote animal welfare (though this remains to be quantified). Such techniques may be particularly valuable for small amphibian species as well as those requiring multiple hormone applications (see below). It is important to note that cutaneous absorption is less efficient than hormone injection and gamete-release responses may be more variable as a consequence. Further research is required before topical or water-bath hormone applications can be used effectively in a wide number of species.

### Frequency of hormone administration to induce reproductive events

At present, research has not been undertaken to determine the stress response of amphibians administered repeat hormone applications or the effect of variation in administration interval on animal welfare. The number of hormone applications required to induce gamete-release in amphibians may be both sex- and species- specific. Female anurans are typically the more difficult sex to hormonally induce gamete release (Clulow *et al*., 2018). Female anurans often require low-dose priming prior to the administration of ovulatory doses of reproductive hormones to elicit the desired ovulatory response, and may require multiple hormone treatments and/or a combination of hormones at short intervals (<48 hours) (Silla, 2011; Silla, 2013; Calatayud *et al*., 2015a; Table 1). In contrast, research to date suggests that anuran sperm release is most effectively induced in response to a single hormone treatment (Silla, 2011; Silla *et al*., 2019; Table 1). Research has shown that male amphibians continue to respond to hormone treatment for up to 24 hours post-administration (Kouba and Vance, 2009; Roth and Obringer, 2003) and that multiple injections within this period can have a negative effect on spermiation (Silla, 2011). Consequently, there appears to be no current rationale for the administration of multiple doses to male anurans. There is some evidence that male urodeles may benefit from priming injections with sperm-release successfully achieved in black-spotted newts (*Notophthalmus meridionalis*), Kweichow newts (*Tylototriton kweichowensis*) and tiger salamanders (*Ambystoma tigrinum*) following the administration of a single priming plus higher spermiation dose of GnRHa (Guy et al., 2020a; Marcec, 2016). Other species, including the Iberian ribbed newt (*Pleurodeles waltl*) and Mexican axolotl (*Ambystoma mexicanum*), respond favourably to a single hormone injection, though the effects of priming injections on the sperm-release response of these species remains untested (Uteshev *et al*., 2015; Mansour *et al*., 2011). To date, research has not been undertaken in either anurans or urodeles to investigate whether there are any benefits to the administration of low-dose priming prior to, or early in the breeding season to stimulate spermatogenesis and improve male fertility, and this warrants investigation.

### Frequency of induced reproductive events annually

Consideration should be given to the frequency of induced reproductive events on an annual cycle. Captive facilities may attempt to induce multiple spawning events within a breeding season or induce spawning out-of-season in order to increase the number of offspring generated annually. There is some evidence that frequent hormone administration can negatively impact spermiation responses and lead to significant weight loss (McDonough *et al*., 2016). In order to maintain sperm concentration and body condition, it has been recommended that male Fowler’s toads (*Anaxyrus (Bufo) fowleri*) be allowed a minimum of 2–3 weeks recovery period between hormone injections (McDonough *et al*., 2016). Recovery periods required for male amphibians to avoid sperm depletion are likely to be species-specific and related to the timing and duration of spermatogenesis, which may differ among species and reproductive modes. Female amphibians should also be provided with species-specific recovery periods to allow females to properly recover energy stores if inducing repeat clutching. Failure to do so may predispose females to secondary infections and reproductive failure (Whitaker, 2001). Females of seasonally breeding amphibian species may require 8–12-month intervals to maintain a high ovulatory response, though a lesser proportion of females may be induced to ovulate after a 4-month recovery period (Guy *et al*., 2020b). In contrast, female South African clawed frogs (*X. laevis*) can be induced to ovulate year-round if provided with a recovery period of only 1–3 months (Green *et al*., 2007). Although extremely rare, frequent, repeat stimulation of ovulation in *X. laevis* has been shown to cause ovarian hyperstimulation syndrome (evidenced by whole-body edema, gross enlargement of the ovaries, ascites and abdominal distention) in some cases (Green et al., 2007). In addition to the importance of allowing a sufficient recovery period between reproductive events annually to maintain reproductive health, it is important to note that there is currently no data available on the costs of multiple breeding events on the long-term health and fitness of amphibians in captivity. Increasing reproductive output by inducing reproduction too frequently could lead to a reduction of the overall reproductive lifespan of an individual (known as reproductive trade-offs, see Stearns, 1992) and this will be an important area for future research. Until species-specific data become available, we suggest that the frequency of induced reproductive events should be limited. Appropriate reproductive intervals should be established, and restoration of body condition (weight, egg production, secondary sex characteristics, etc.) between breeding events should be carefully monitored. This may also be important for ensuring offspring fitness, for example if females are in poor condition, yolk provisioning will be compromised, which is known to have a strong influence on offspring size (see Dziminski and Alford, 2005). We recommend that the natural reproductive intervals of species’ (e.g. the average number of spawning events observed per individual, per breeding season, within wild populations) should be replicated to avoid over stimulating reproductive effort beyond natural levels. This will be particularly important for females as the cost of reproduction is generally much higher in the female sex (Stearns, 1992). Knowledge of a species’ natural reproductive ecology is vital for successful conservation breeding programs and every effort should be made to gain this information and refine protocols accordingly.

### State of amphibian reproductive technologies for induced gamete release and spawning

Amphibians have been used as laboratory models for the study of developmental embryology for more than a century, facilitating a vast number of scientific discoveries associated with gene expression, cloning, stem cell pluripotency and epigenesis, so these technologies are not novel (Silla and Byrne 2019). However, the application of reproductive technologies beyond a small number of model species has been achieved only in the past 40-years and these technologies have only recently begun to be recognized as an invaluable tool for amphibian conservation (Clulow *et al*., 2014; Kouba *et al*., 2013; Silla and Byrne, 2019). Protocols for successfully inducing gamete release in amphibians have been reviewed previously and we direct the reader to comprehensive summaries published elsewhere to complement the information provided herein (Bronson and Vance, 2019; Clulow *et al*., 2014; Kouba *et al*., 2013; Silla and Byrne, 2019; Vu and Trudeau, 2016; Whitaker, 2001). Briefly, several forms of synthetic gonadotropin-releasing hormone agonists (GnRH-a, also known as luteinizing hormone-releasing hormone agonist and LHRH-a) and purified human chorionic gonadotropin (hCG) are the most commonly used exogenous hormones administered to promote spawning in pairs of amphibians, or to stimulate ovulation in females and spermiation in males for separate gamete collection for IVF (Table 1). A GnRH-a acts at the level of the pituitary to stimulate the synthesis and release of both LH and FSH from the anterior pituitary (Fig. 1). Human chorionic gonadotropin is an agonist of LH, and injection attempts to mimic the LH surge required to stimulate ovulation or sperm release (see see Part 1: *Hormonal control of Reproduction* and Fig. 1). GnRH-a is generally regarded as more effective for inducing spawning and gamete release in a diversity of amphibians, however there are a number of species where spermiation is achieved more successfully in response to the administration of hCG, including several species from the Bufonid, Limnodynastid and Pelodryadid families (Kouba *et al*., 2012a; Clulow *et al*., 2018; Silla and Roberts, 2012; Silla *et al*., 2019). The variation in response to hCG is driven by species-specific differences in gonadal LH-receptor affinities. Optimal doses required to induce spawning or gamete release have also been shown to vary enormously among amphibian species (Table 1), highlighting the importance of establishing optimal dosages for a given species, and to search for more effective gonadotropin preparations and alternative strategies. In addition to species-specific differences in optimal hormone therapies, amphibians also display sex-specific variation in responses (Silla, 2011; Clulow *et al*., 2018). In general, gamete release is more difficult to achieve in females compared to males (Clulow *et al*., 2018). Sperm release is achieved in most male amphibians (with the exception of some male urodeles e.g. see Guy *et al*., 2020a) via the administration of a single hormone and dose, while female conspecifics often require low-dose priming injections prior to the administration of a higher ovulatory dose and/or a combination of hormone types to achieve successful ovulation (Table 1). Emerging evidence suggests that the neurotransmitter DA can inhibit LH release in some species. Combined injection of a GnRH-a in combination with a DA antagonist (e.g. domperidone or metoclopramide), an approach named the AMPHIPLEX method, has been successful at inducing spawning in a number of species (Trudeau et al., 2010; Della Togna *et al*., 2017; Clulow *et al*., 2018; Clulow *et al*., 2019). Essentially, a DA antagonist blocks dopaminergic inhibition, to enhance GnRH-stimulated LH release, generating an LH surge, thus inducing spawning (Fig. 1). There are likely species differences in the relative importance of DA for the control of LH release, as has been observed in teleosts. Although some mechanistic data is available (Vu *et al*., 2017) there is a clear need for more research.

Protocols for the hormonal induction of gamete release are often used in concert with subsequent reproductive technologies that further enhance the genetic management of threatened species. Comprehensive reviews of short-term gamete storage (Silla and Byrne, 2019; Browne *et al*., 2019), sperm cryopreservation and biobanking of genetic material (Clulow *et al*., 2014, 2019; Browne *et al*., 2019; Della Togna et al., 2020), and IVF (Kouba *et al*., 2009; Silla and Byrne, 2019) protocols are available elsewhere and will not be specifically addressed here.

## Part 4: Case studies

### Mountain yellow-legged frog

The mountain yellow-legged complex is comprised of two distinct species, *R. muscosa* and *Rana sierrae*, which are both endemic to California, USA. Since the 1970’s, *R. muscosa* has declined from 166 reported locations in Southern California to only 8 (Hammerson, 2008; Backlin *et al*., 2015) and is classified as endangered by the IUCN. Reasons for decline typify the problem globally attributed to the combined impact of loss of habitat, introduction of invasive exotic species (particularly fish) and chytrid fungus (*Bd*) (Backlin *et al*., 2015). Mountain yellow-legged frogs breed from early spring (April at lower elevations), at the onset of ice-melting in the mountains or June–July (at higher elevations) (Stebbins, 1951). The species is sexually dimorphic, females are larger (snout-vent-length (SVL), ~70 mm) than males (SVL, ~60 mm) (Backlin *et al*. 2015) and sexual maturity in the wild reportedly occurs between 3 and 5 years of age (with males typically reaching sexual maturity 1 to 2 years faster than females). Average clutch size is ~230 eggs (https://amphibiaweb.org).

### Captive breeding and reintroduction program

San Diego Zoo’s Global-Beckman Center for Conservation Research is the largest captive breeder of *R. muscosa* currently holding more animals in captivity than are estimated to remain in the wild (Backlin, *et al*., 2015). The conservation program began in 2006, after 82 tadpoles and 2 egg masses were rescued from a drying creek during a salvage operation and transferred to the San Diego Zoo for a head-start program.

In captivity, breeding was first observed in 2008 (when founders were only 2 years old) when 1 of the 25 females deposited a clutch of 100 eggs. From this first clutch only one individual survived metamorphosis to become a breeding male. In 2009, no females oviposited, and poor reproduction was attributed to females not reaching sexual maturity or a lack of brumation (Santana *et al*., 2015). Therefore, in the winter of 2010–2011, frogs were brumated prior to the breeding season and 21 females oviposited. Over the past decade, the number of females ovipositing and clutch sizes (average clutch size is 468 eggs/clutch) in captivity have increased but continue to vary dramatically, while the number of eggs laid that successfully cleaved as embryos after fertilization increased from 3% in the first year to 80–86% 4 years later (Calatayud *et al*., 2015b, 2016; Gardner *et al*., 2018). In 2012, the animals in the colony had all reached sexual maturity and this was reflected in an increase in reproductive output. Survival rates from embryos to tadpoles also increased yearly averaging 74%. From 2015 onwards, a proportion of tadpoles (~200/year) were raised in captivity and reintroduced as 1st year froglets in the subsequent year (Calatayud *et al*., 2015b). The observed increased oviposition may be explained by an increase in the number of sexually mature females, a biennial or triennial reproductive cycle in this species. However, no data exists in the wild on whether *R. muscosa* breed annually or if they may skip a breeding season. Alternatively, improved nutrition could also be linked to increasing numbers of ovipositing females from 2014 to 2016. Such speculations emphasize the need for directed research in this area.

### Incorporating reproductive technologies

**Figure 2:**
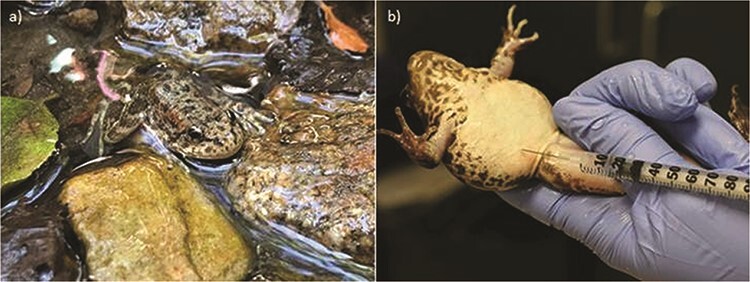
(a) Mountain yellow-legged frog, *R. muscosa* (photo credit: Tali Hammond); (b) administration of hormones by intraperitoneal injection to an adult female (image taken from Calatayud et al., 2019 with permission from the *Journal of Visual Experimentation*).

Injection of exogenous hormones for *R. muscosa* captive breeding program started in 2013 (Fig. 2b). Administration of a GnRH-a, DA antagonist metoclopramide, prostaglandin (PGF2α) and the gonadotropin hCG have all been tested with the goal of increasing reproductive behaviours, obtaining gametes for IVF and cryopreservation and inducing oviposition in females that have retained eggs (Calatayud *et al*., 2019). However, because *R. muscosa* can spend a long time in amplexus, from 8 days to 4 weeks, before oviposition occurs, it is difficult to predict the most appropriate time to inject animals and to measure the efficacy of hormone therapy (Calatayud *et al*., 2015b). Compared to control animals, reproductive behaviours do not significantly increase in *R. muscosa* breeding pairs nor did the percentage of eggs produced and fertilized after administration of various hormone preparations (Calatayud *et al*., 2015b). However, it is important to note that it can be difficult to determine which animals are preovulatory, a generalized problem for captive breeding in amphibians, unless husbandry practices incorporate other reproductive technologies such as ultrasound and hormone monitoring into their captive protocols (Calatayud *et al*., 2019; Nagel *et al*., 2019). When examining spermiation independently, both GnRH-a and hCG successfully stimulated the release of sperm by males with GnRH-a being more effective than hCG (Gardner et al., 2018; Calatayud, unpublished data). Induction of spermiation seems to be associated with breeding (end of March to June) and pre-brumation (October and November) sperm production and release as reported in other high elevation temperate anurans (Jorgensen, 1992; Calatayud, unpublished data).

To date, the captive *R. muscosa* population has been capable of breeding without hormone injection, though spawning is unpredictable and inconsistent. There are data to suggest that this species does not spawn every year, which adds to the challenges of controlling reproduction in captivity. There are other factors that may contribute to low reproductive outputs, such as, a potentially reduced genetic diversity of the colony (Cynthia Steiner pers. comm.) and several cases of hermaphroditism in the founding population (Leah Jacobs pers. comm.). However, since 2015, increases in reproductive outputs may likely relate to changes in husbandry practises including increased diversity of food items, optimization of laboratory temperature and light cycles, water quality testing and maintenance and water temperature manipulation for breeding. Brumation has not only been shown to be a reproductive necessity (Santana *et al*., 2015) but also an influential factor in the survival of animals released back into the wild (Calatayud *et al*., 2020). To date, over 6000 tadpoles and approximately 1000 froglets have been head started and released into the wild. Studies to improve spawning success, increase tadpole and froglet numbers, improve genetic diversity and understand the microbiome of captive versus released individuals in the breeding population are underway.

### Northern corroboree frog

**Figure 3:**
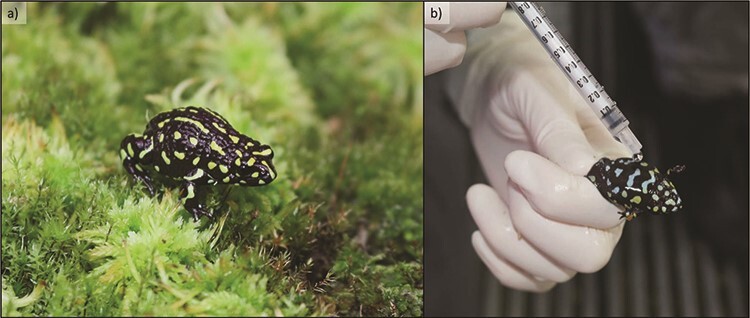
(a) Male northern corroboree frog, *P. pengilleyi*; (b) topical application of reproductive hormones by syringe application of drops on the ventral surface of *P. pengilleyi*. The topical application of a GnRH-a in 2017 generated in excess of 300 viable embryos from 18 clutches. Incorporating reproductive technologies into the existing captive breeding and reintroduction program for the northern corroboree frog will allow better control over the genetic management of the species, including the selective breeding of particular genotypes. Photo credits: Aimee Silla.

The northern corroboree frog (*P. pengilleyi*) is a small (snout–vent length, 25–30 mm), terrestrial frog easily recognized by its striking black and lime-green/yellow colouration (Fig. 3a). The species is restricted to alpine habitats above 850 m altitude in the Brindabella and Fiery ranges of New South Wales and the Australian Capital Territory in south-eastern Australia. Early observations document the species in great abundance (Colefax, 1956), though the species has suffered severe and rapid population declines since the mid-1980s due primarily to the spread of the amphibian chytrid fungus (*Bd*) (Hunter *et al*., 2010). The species is listed as critically endangered by state and federal governments and is listed as endangered by the IUCN (McFadden *et al*., 2016).

Breeding in this species commences in late austral summer and continues until early autumn. Male *P. pengilleyi* construct shallow terrestrial nests where females oviposit a small clutch of between 16 and 40 (mean = 24.0) eggs, which are externally fertilized (Osborne, 1991). Fertilized eggs undergo intracapsular embryonic development, which is typically suspended at Gosner Stages 26–28. Terrestrial embryos may remain in suspended development for several weeks until heavy winter rainfall floods the nest and hypoxia triggers tadpoles to hatch into temporary pools (Osborne, 1991). This reproductive mode (terrestrial egg mass with aquatic larvae) is characteristic of the genus *Pseudophryne*.

### Captive breeding and reintroduction program

An insurance colony was established for the Northern corroboree frog at the Tidbinbilla Nature Reserve in the Australian Capital Territory, following the annual collection of fertilized eggs from wild populations between 2003 and 2008 (Holz *et al*., 2015). A substantial number of animals were subsequently transferred to Taronga Zoo, Sydney and a collaborative captive breeding and reintroduction program has been established from a partnership between the two captive institutions and the NSW Office of Environment and Heritage (McFadden *et al*., 2016). Captive breeding protocols have been established with the first viable clutch of eggs laid in captivity in 2008 (Holz *et al*., 2015). As with its sister species, the Southern corroboree frog (*P. corroboree*), key requirements for achieving successful breeding of this species include adequate temperature cycling that incorporates a winter cooling/brumation period prior breeding, and the provision of large breeding tanks containing multiple males to allow for female mate-choice (McFadden *et al*., 2013).

### Incorporating reproductive technologies

Although the northern corroboree frog has been bred successfully in captivity for a number of years, captive populations display strong mating bias with less than a third of available males contributing to mating success annually. The natural mating system of this species has similarly been shown to exhibit strong mating bias towards a small number of male individuals, so this mating pattern is not unique among captive populations. However, over time, such captive mating biases may lead to a loss of genetic variation in the offspring generated for re-introduction. Between 2014 and 2017, experimental trials were conducted as part of a strategic research collaboration between the University of Wollongong and Taronga Conservation Society Australia, employing hormone-induced spawning protocols to assist the genetic management of the species. Administration of a single injection per animal of GnRH-a, was used to induce spawning in isolated male–female pairs housed together in small aquaria. Hormone administration significantly improved the number of pairs producing viable embryos (from 22% to 100%) and total egg number (Silla *et al*., 2018). Overall, in excess of 800 viable eggs (from 37 clutches) were generated using hormonal induction between 2014 and 2016, which were subsequently released into natural sites in the Northern Brindabella Ranges. Following on from initial experiments, in 2017 the research team developed successful techniques for the topical application of reproductive hormones to induce breeding in the northern corroboree frog. Applying reproductive hormones topically eliminates the need for injection, which has the potential to improve animal welfare, though this remains to be quantified. Additionally, topical hormone administration protocols are cost-effective for small species and eliminate the need for specialized training in animal injection or the need for large quantities of single-use sterile needles, which may allow reproductive technologies to be more widely adopted by amphibian captive breeding facilities globally. It is important to note that research on topical application of reproductive hormones to induce spawning or gamete release in amphibians is in its infancy, so much work remains.

## Part 5: Conclusions

With over 8100 described species of amphibians recorded in the world and ~41% threatened with extinction (categorized as critically endangered, endangered or vulnerable) (IUCN 2020), establishing captive breeding programs for endangered species is increasingly important. Reproductive technologies have enormous potential to contribute to breeding programs by manipulating neuroendocrine systems in order to circumvent the behavioural and physical impediments to natural reproduction exhibited by amphibians in both short- and long-term captivity. When traditional captive breeding methods (manipulating environmental conditions) are used in concert with reproductive technologies, outcomes are likely to be most successful.

Little is known about the factors that inhibit reproduction. Handling and chronic husbandry conditions that inappropriately activate the HPI axis, leading to CORT-mediated inhibition of reproduction must be mitigated. However, basic knowledge of the endocrinology of reproduction, growth and stress of amphibians is very poorly understood, impeding the development of novel hormone therapies to promote gamete release and spawning. Amphibians exhibit both species-specific and sex-specific differences in their responses to hormone treatments. There is the additional challenge in that amphibians exhibit a diverse array of reproductive strategies, and although speculative, it is probable that neuroendocrine control mechanisms may also be more diverse than currently assumed. Application of the general principles of comparative and evolutionary endocrinology is the starting point, but significant species differences exist. It will be critical that research groups collaborate to identify and target representative species with differing reproductive strategies to advance our knowledge strategically. Critical to the success of a breeding program is the accurate identification and selection of mature animals, so research efforts should be placed on non-invasive assessment methods (e.g. ultrasound, assessment of waterborne, urinary, and salivary sex steroid levels), so that inappropriate activation of the HPI axis (e.g. excess CORT) can be avoided. It will be very important to validate antibody-based steroid hormone detection methods (e.g. radioimmunoassays and enzyme-linked immunoassays) adequately or to switch to more sensitive methods (albeit more expensive) such as liquid chromatography and mass spectrometry since few have been specifically developed for the detection of waterborne and urinary steroids in amphibians. Moreover, conjugation of steroids for excretion from the body is poorly studied in amphibians and will require significant efforts for identification and measurement using state-of-the-art mass spectrometry (Lee *et al*., 2020; Son *et al*., 2020). Nevertheless, there is an increasing number of species that can be bred in captivity and managed effectively with the aid of reproductive technologies. Further research is required to develop novel hormone-induction protocols for a greater number of threatened amphibian species to expedite the incorporation of reproductive technologies into captive breeding programs to boost global conservation efforts.

## Funding

We acknowledge the following funding sources: Australian Research Council (LP140100808 & DE210100812) and NSW Environmental Trust to A.J.S.; Exportadora de Immuebles S.A. and San Diego Zoo post-doctoral program to N.E.C.; and Environment and Climate Change Canada, Ministère Forêt Faune et Parcs Québec, NSERC-Canada, University of Ottawa Research Chair Program to V.L.T.

## References

[ref1] Asa C, Traylor-Holzer K, Lacy R (2011) Can conservation-breeding programmes be improved by incorporating mate choice? Int Zoo Yearb 45: 203–212.

[ref2] Backlin AR, Hitchcock CJ, Gallegos EA, Yee JL, Fisher RN (2015) The precarious persistence of the endangered Sierra Madre yellow-legged frog *Rana muscosa* in southern California, USA. Oryx 49: 157–164.

[ref3] Ball J (1981) Hypothalamic control of the pars distalis in fishes, amphibians, and reptiles. Gen Comp Endocrinol 44: 135–170. 6265311 10.1016/0016-6480(81)90243-4

[ref4] Bronson E, Vance CK (2019) Anuran reproduction) Fowler's Zoo and Wild Animal Medicine Current Therapy, vol. 9. Elsevier, St. Louis, Missouri, pp. 371–379

[ref5] Browne R, Li H, Seratt J, Kouba A (2006) Progesterone improves the number and quality of hormone induced fowler toad (Bufo fowleri) oocytes. Reprod Biol Endocrinol 4:3. 10.1186/1477-7827-4-3 PMC1373633 16451718

[ref6] Browne RK, Silla AJ, Upton R, Della-Togna G, Marcec-Greaves R, Shishova NV, Uteshev VK, Proaño B, Pérez OD, Mansour N et al. (2019) Sperm collection and storage for the sustainable management of amphibian biodiversity. Theriogenology 133: 187–200. 31155034 10.1016/j.theriogenology.2019.03.035

[ref7] Berger L, Roberts AA, Voyles J, Longcore JE, Murray KA, Skerratt LF (2016) History and recent progress on chytridiomycosis in amphibians. Fung Ecol 19: 89–99.

[ref8] Borah BK, Renthlei Z, Trivedi AK (2019) Seasonality in terai tree frog (Polypedates teraiensis): role of light and temperature in regulation of seasonal breeding. J Photochem Photobiol 191: 44–51. 10.1016/j.jphotobiol.2018.12.005 30580184

[ref9] Byrne PG, Gaitan Espitia JD, Silla AJ (2019) Genetic benefits of extreme sequential polyandry in a terrestrial-breeding frog. Evolution 73: 1972–1985. 31411350 10.1111/evo.13823

[ref10] Byrne PG, Roberts JD (2012) Evolutionary causes and consequences of sequential polyandry in anuran amphibians. Biol Rev 87: 209–228. 21740503 10.1111/j.1469-185X.2011.00191.x

[ref11] Byrne PG, Silla AJ (2020) An experimental test of the genetic consequences of population augmentation in an amphibian. Conserv Sci Pract 2: e194.

[ref12] Calatayud NE, Gardner N, Shier DM (2016) Captive breeding and reintroduction of the mountain yellow-legged frog (*Rana muscosa*). Annual Report, San Diego Zoo Institute for Conservation Research, USA, p. 2016.

[ref13] Calatayud NE, Langhorne CJ, Mullen AC, Williams CL, Smith T, Bullock L, Kouba AJ, Willard ST (2015a) A hormone priming regimen and hibernation affect oviposition in the boreal toad *(Anaxyrus boreas boreas)*. Theriogenology 84: 600–607. 26025241 10.1016/j.theriogenology.2015.04.017

[ref14] Calatayud NE, Gardner N, Shier DM (2015b) Captive breeding and reintroduction of the mountain yellow-legged frog (*Rana muscosa*): 2015 Annual Report, San Diego Zoo Institute for Conservation Research, USA.

[ref15] Calatayud NE, Stoops M, Durrant BS (2018) Ovarian control and monitoring in amphibians. Theriogenology 109: 70–81. 29325879 10.1016/j.theriogenology.2017.12.005

[ref16] Calatayud NE, Chai N, Gardner NR, Curtis MJ, Stoops MA (2019) Reproductive techniques for ovarian monitoring and control in amphibians. J Vis Exp 147: e58675. doi: 10.3791/58675. 31132048

[ref17] Calatayud NE, Hammond TT, Gardner NR, Curtis MJ, Swaisgood RR, Shier DM (2020) Benefits of overwintering in the conservation breeding and translocation of a critically endangered amphibian. Conserv Sci Pract 2020: e341.

[ref18] Carey C, Corn PS, Jones MS, Livo LJ, Muths E, Loeffler CW (2005) Factors limiting the recovery of boreal toads *(Bufo b. boreas)*. In Amphibian Declines: The Conservation Status of United States Species, University of California Press, Berkeley and Los Angeles, California. pp. 222–236.

[ref19] Cayuela H, Léna J-P, Lengagne T, Kaufmann B, Mondy N, Konecny L, Dumet A, Vienney A, Joly P (2017) Relatedness predicts male mating success in a pond-breeding amphibian. Anim Behav 130: 251–261.

[ref20] Chabbi A, Ganesh C (2015) Evidence for the involvement of dopamine in stress-induced suppression of reproduction in the cichlid fish *Oreochromis mossambicus*. J Neuroendocrinol 27: 343–356. 25712855 10.1111/jne.12269

[ref21] Chand D, Lovejoy DA (2011) Stress and reproduction: controversies and challenges. Gen Comp Endocrinol 171: 253–257. 21362426 10.1016/j.ygcen.2011.02.022

[ref22] Chieffi G, Rastogi RK, Milone M, Iela L (1980) Amphibian reproduction: reproductive physiology in the male Rana esculenta L. Ital J Zool 47: 63–70.

[ref24] Clulow J, Pomering M, Herbert D, Upton R, Calatayud N, Clulow S, Mahony MJ, Trudeau VL (2018) Differential success in obtaining gametes between male and female australian temperate frogs by hormonal induction: a review. Gen Comp Endocrinol 265: 141–148. 29859744 10.1016/j.ygcen.2018.05.032

[ref25] Clulow J, Trudeau VL, Kouba AJ (2014) Amphibian declines in the twenty-first century: why we need assisted reproductive technologies. Adv Exp Med Biol 753: 275–316. 25091914 10.1007/978-1-4939-0820-2_12

[ref26] Clulow J, Upton R, Trudeau VL, Clulow S (2019) Amphibian assisted reproductive technologies: moving from technology to application. Adv Exp Med Biol 1200: 413–463. 31471805 10.1007/978-3-030-23633-5_14

[ref27] Cockrem JF (2013) Individual variation in glucocorticoid stress responses in animals. Gen Comp Endocrinol 181: 45–58. 23298571 10.1016/j.ygcen.2012.11.025

[ref28] Coddington E, Cree A (1995) Effect of acute captivity stress on plasma concentrations of corticosterone and sex steroids in female whistling frogs, *Litoria ewingi*. Gen Comp Endocrinol 100: 33–38. 8575656 10.1006/gcen.1995.1129

[ref29] Colefax A (1956) New information on the corroboree frog *(Pseudophryne corroboree* Moore). Linnean Society of New South Wales 80: 258–266.

[ref30] Creighton AE, Wilczynski W (2014) Influence of dopamine D2-type receptors on motor behaviors in the green tree frog. Hyla cinerea. Physiol Behav 127: 71–80. 24480075 10.1016/j.physbeh.2014.01.005

[ref31] Czuchlej SC, Volonteri MC, Regueira E, Ceballos NR (2019) Effect of glucocorticoids on androgen biosynthesis in the testes of the toad *Rhinella arenarum* (Amphibia, Anura). *Journal of Experimental Zoology Part A: Ecological and Integrative*. Phys Ther 331: 17–26. 10.1002/jez.2232 30218550

[ref32] Davis A, Abraham E, McEvoy E, Sonnenfeld S, Lewis C, Hubbard CS, Dolence EK, Rose JD, Coddington E (2015) Corticosterone suppresses vasotocin-enhanced clasping behavior in male rough-skinned newts by novel mechanisms interfering with V1a receptor availability and receptor-mediated endocytosis. Horm Behav 69: 39–49. 25528549 10.1016/j.yhbeh.2014.12.006

[ref33] de Assis VR, Navas CA, Mendonça MT, Gomes FR (2012) Vocal and territorial behavior in the Smith frog *(Hypsiboas faber):* relationships with plasma levels of corticosterone and testosterone. Comp Biochem Physiol A Mol Integr Physiol 163: 265–271. 22903053 10.1016/j.cbpa.2012.08.002

[ref34] Della Togna G, Trudeau VL, Gratwicke B, Evans M, Augustine L, Chia H, Bronikowski EJ, Murphy JB, Comizzoli P (2017) Effects of hormonal stimulation on the concentration and quality of excreted spermatozoa in the critically endangered Panamanian golden frog (*Atelopus zeteki*). Theriogenology 91: 27–35. 28215683 10.1016/j.theriogenology.2016.12.033

[ref156] Della Togna G, Howell LG, Clulow J, Langhorne CJ, Marcec-Greaves R, Calatayud NE (2020) Evaluating amphibian biobanking and reproduction for captive breeding programs according to the Amphibian Conservation Action Plan objectives. Theriogenology 150: 412–431. 32127175 10.1016/j.theriogenology.2020.02.024

[ref35] Denari D, Ceballos NR (2005) 11β-Hydroxysteroid dehydrogenase in the testis of Bufo arenarum: changes in its seasonal activity. Gen Comp Endocrinol 143: 113–120. 16061069 10.1016/j.ygcen.2005.03.006

[ref36] Di Fiore MM, Santillo A, Falvo S, Pinelli C (2020) Celebrating 50+ years of research on the reproductive biology and endocrinology of the green frog: an overview. Gen Comp Endocrinol 298: 113578. 32739437 10.1016/j.ygcen.2020.113578

[ref37] Dufour S, Sebert ME, Weltzien FA, Rousseau K, Pasqualini C (2010) Neuroendocrine control by dopamine of teleost reproduction. J Fish Biol 76: 129–160. 20738703 10.1111/j.1095-8649.2009.02499.x

[ref38] Dufour S, Weltzien FA, Sebert ME, Le Belle N, Vidal B, Vernier P, Pasqualini C (2005) Dopaminergic inhibition of reproduction in teleost fishes: ecophysiological and evolutionary implications. Ann N Y Acad Sci 1040: 9–21. 15891002 10.1196/annals.1327.002

[ref39] Dugas MB, Yeager J, Richards-Zawacki CL (2013) Carotenoid supplementation enhances reproductive success in captive strawberry poison frogs (Oophaga pumilio). Zoo Biol 32: 655–658. 24151130 10.1002/zoo.21102

[ref40] Dziminski MA, Vercoe PE, Roberts JD (2009) Variable offspring provisioning and fitness: a direct test in the field. Funct Ecol 23: 164–171.

[ref41] Dziminski MA, Alford RA (2005) Patterns and fitness consequences of intraclutch variation in egg provisioning in tropical Australian frogs. Oecologia 146: 98–109. 16003504 10.1007/s00442-005-0177-2

[ref42] Eisthen HL, Krause BC (2012) Ambiguities in the relationship between gonadal steroids and reproduction in axolotls (Ambystoma mexicanum). Gen Comp Endocrinol 176: 472–480. 22245262 10.1016/j.ygcen.2011.12.034

[ref43] Ferrie GM, Alford VC, Atkinson J, Baitchman E, Barber D, Blaner WS, Crawshaw G, Daneault A, Dierenfeld E, Finke M (2014) Nutrition and health in amphibian husbandry. Zoo Biol 33: 485–501. 25296396 10.1002/zoo.21180 PMC4685711

[ref45] Fitzgeral SD, Duncan AE, Tabaka C, Garner MM, Dieter A, Kiupel M (2007) Ovarian dysgerminomas in two mountain chicken frogs (*Leptodactylus fallax*). J Zoo Wildl Med 38: 150–153. 17469294 10.1638/06-015.1

[ref44] Gardner N, Calatayud N, Shier DM (2018). Captive breeding and reintroduction of the mountain yellow-legged frog (*Rana muscosa*): 2017. Annual Report, San Diego Zoo Institute for Conservation Research, USA, p. 2018.

[ref46] Gascon C, Collins JP, Moore RD, Church DR, McKay JE, Mendelson III JR (2007) Amphibian Conservation Action Plan. London, IUCN SSC Amphibian Specialist Group, http://www.amphibians.org/wpcontent/uploads/2013/07/ACAP.pdf

[ref47] Gracia-Navarro F, Licht P (1987) Subcellular localization of gonadotrophic hormones LH and FSH in frog adenohypophysis using double-staining immunocytochemistry. J Histochem Cytochem 35: 763–769. 3108366 10.1177/35.7.3108366

[ref48] Graham KM, Langhorne CJ, Vance CK, Willard ST, Kouba AJ (2018) Ultrasound imaging improves hormone therapy strategies for induction of ovulation and in vitro fertilization in the endangered dusky gopher frog (*Lithobates sevosa*). Conserv Physiol 6: coy020. 10.1093/conphys/coy020 PMC5925431 29732159

[ref49] Green SL, Parker J, Davis C, Bouley DM (2007) Ovarian hyperstimulation syndrome in gonadotropin-treated laboratory south African clawed frogs *(Xenopus laevis)*. J Am Assoc Lab Anim Sci 46: 64–67. 17487957

[ref50] Griffiths RA, Garcia G, Oliver J (2008) Re-introduction of the Mallorcan midwife toad, Mallorca, Spain. Global Re-introduction Perspectives: 2008 Case-studies from around the globe IUCN/SSC Re-introduction Specialist Group, 54–57.

[ref51] Gupta BK, Tapley B, Vasudevan K, Goetz M (2015) Ex situ management of amphibians. Assam State Zoo Cum Botanical Garden. Guwahati, Assam, India.

[ref155] Guy EL, Gillis AB, Kouba AJ, Barber D, Poole B, Marcec-Greaves RM, Kouba CK (2020a) Sperm collection and cryopreservation for threatened newt species. Cryobiology 94: 80–88. 32437677 10.1016/j.cryobiol.2020.04.005

[ref52] Guy EL, Martin MW, Kouba AJ, Cole JA, Kouba CK (2020b) Evaluation of different temporal periods between hormone-induced ovulation attempts in the female Fowler’s toad Anaxyrus fowleri. Conserv Physiol 8: coz113. 10.1093/conphys/coz113 PMC6951360 31938544

[ref53] Haddad CFB, Prado CPA (2005) Reproductive modes in frogs and their unexpected diversity in the Atlantic forest of Brazil. Bioscience 55: 207–217.

[ref54] Hammerson G (2008). *Rana muscosa*. The IUCN Red List of Threatened Species 2008: e.T19177A8847938. 10.2305/IUCN.UK.2008.RLTS.T19177A8847938.en. Downloaded on 02 September 2019.

[ref55] Holz P, Portas T, Donahoe S, Crameri S, Rose K (2015) Mortality in northern corroboree frog tadpoles *(Pseudophryne pengilleyi*) associated with Tetrahymena-like infection. Aust Vet J 93: 295–297. 26220324 10.1111/avj.12337

[ref56] Houck LD, Mendonça MT, Lynch TK, Scott DE (1996) Courtship behavior and plasma levels of androgens and corticosterone in male marbled salamanders, *Ambystoma opacum* (Ambystomatidae). Gen Comp Endocrinol 104: 243–252. 8930615 10.1006/gcen.1996.0167

[ref57] Hunter DA, Speare R, Marantelli G, Mendez D, Pietsch R, Osborne W (2010) Presence of the amphibian chytrid fungus *Batrachochytrium dendrobatidis* in threatened corroboree frog populations in the Australian Alps. Dis Aquat Organ 92: 209–216. 21268983 10.3354/dao02118

[ref58] Hussain QA (2012) Global amphibian declines: a review. Int J Biodivers Conserv 4: 348–357.

[ref59] IUCN (2020) Table 1a: number of species evaluated in relation to overall number of described species, and numbers of threatened species by major groups of organisms. IUCN Red List Version 2020–2022. http://www.iucnredlist.org.

[ref60] Jorgensen C (1992) Growth and reproduction. In. Feder ME, Burggren WW (eds). In Environmental Physiology of the Amphibians, University of Chicago Press, pp 439–466

[ref61] Joshi A, Narayan EJ, Gramapurohit NP (2018) Interrelationship among annual cycles of sex steroids, corticosterone and body condition in *Nyctibatrachus humayuni*. Gen Comp Endocrinol 260: 151–160. 29339182 10.1016/j.ygcen.2018.01.013

[ref62] Kaiser K, Devito J, Jones CG, Marentes A, Perez R, Umeh L, Weickum RM, McGovern KE, Wilson EH, Saltzman W (2015) Reproductive and immune effects of chronic corticosterone treatment in male White’s treefrogs, Litoria caerulea. Conserv Physiol 3: cov022. 10.1093/conphys/cov022 PMC4778456 27293707

[ref63] Keogh LM, Byrne PG, Silla AJ (2015) The effect of gentamicin on sperm motility and bacterial abundance during chilled sperm storage in the Booroolong frog. Gen Comp Endocrinol 243: 51–59. 10.1016/j.ygcen.2016.11.005 27823953

[ref64] Keogh LM, Byrne PG, Silla AJ (2018a) Effect of long-term dietary beta-carotene supplementation on sperm concentration and motility in an endangered amphibian. Anim Reprod Sci 195: 259–265. 31262404 10.1016/j.anireprosci.2018.06.003

[ref65] Keogh LM, Silla AJ, McFadden MS, Byrne PG (2018b) Dose and life stage-dependent effects of dietary beta-carotene supplementation on the growth and development of the Booroolong frog. Conserv Physiol 6: coy052. 10.1093/conphys/coy052 PMC6144775 30254750

[ref66] Kouba AJ, Delbarco-Trillo J, Vance CK, Milam C, Carr M (2012a) A comparison of human chorionic gonadotropin and luteinizing hormone releasing hormone on the induction of spermiation and amplexus in the American toad (*Anaxyrus americanus)*. Reprod Biol Endocrinol 10: 59. 22905699 10.1186/1477-7827-10-59 PMC3495228

[ref67] Kouba AJ, Vance CK, Calatayud N, Rowlison T, Langhorne C, Willard S (2012b) Assisted reproductive technologies (ART) for amphibians. Amphibian Husbandry Resource Guide, Edition 2 60–118.

[ref68] Kouba AJ, Lloyd RE, Houck ML, Silla AJ, Calatayud N, Trudeau VL, Clulow J, Molinia F, Langhorne C, Vance C et al. (2013) Emerging trends for biobanking amphibian genetic resources: the hope, reality and challenges for the next decade. Biol Conserv 164: 10–21.

[ref69] Kouba A, Vance C, Willis E (2009) Artificial fertilization for amphibian conservation: current knowledge and future considerations. Theriogenology 71 (1):214–227. 19026442 10.1016/j.theriogenology.2008.09.055

[ref70] Kouba AJ, Vance CK (2009) Applied reproductive technologies and genetic resource banking for amphibian conservation. Reprod Fertil Dev 21: 719–737. 19567216 10.1071/RD09038

[ref71] Kraaijeveld-Smit FJ, Griffiths RA, Moore RD, Beebee TJ (2006) Captive breeding and the fitness of reintroduced species: a test of the responses to predators in a threatened amphibian. J Appl Ecol 43: 360–365.

[ref72] Kubokawa K, Ishii S (1980) Follicle-stimulating hormone (FSH) receptors in the testis of the newt, *Cynops pyrrhogaster*, and comparison of temperature dependency of the receptors with those of the other vertebrates. Gen Comp Endocrinol 40: 425–433. 6245987 10.1016/0016-6480(80)90005-2

[ref73] Kubokawa K, Ishii S (1987) Receptors for native gonadotropins in amphibian liver. Gen Comp Endocrinol 68: 260–270. 2828151 10.1016/0016-6480(87)90037-2

[ref74] Lee YR, Im E, Kim H, Lew BL, Sim WY, Lee J, Oh HB, Paeng KJ, Hong J, Chung BC (2020). Untargeted metabolomics and steroid signatures in urine of male pattern baldness patients after Finasteride treatment for a year. Metabolites.;10: pii: E131. 10.3390/metabo10040131 PMC7241081 32235609

[ref154] Lehman GC (1977) Environmental influence on ovulation and embryonic development in *Rana pipiens*. Journal of Experimental Zoology 199: 51–56. 10.1002/jez.1401990107 300097

[ref75] Lentini A (2007) Husbandry Manual Puerto Rican Crested Toad (*Peltophryne lemur*), 2006/07 Update. Keeper and Curator Edition. Toronto Zoo, Canada.

[ref76] Licht P (1979) Reproductive endocrinology of reptiles and amphibians: gonadotropins. Annu Rev Physiol 41: 337–351. 373595 10.1146/annurev.ph.41.030179.002005

[ref77] Licht P, McCreery BR, Barnes R, Pang R (1983) Seasonal and stress related changes in plasma gonadotropins, sex steroids, and corticosterone in the bullfrog, *Rana catesbeiana*. Gen Comp Endocrinol 50: 124–145. 6406295 10.1016/0016-6480(83)90249-6

[ref78] Licht P, Tsai P-S, Sotowska-Brochocka J (1994) The nature and distribution of gonadotropin-releasing hormones in brains and plasma of ranid frogs. Gen Comp Endocrinol 94: 186–198. 7926629 10.1006/gcen.1994.1075

[ref79] Madelaire CB, Gomes FR (2016) Breeding under unpredictable conditions: annual variation in gonadal maturation, energetic reserves and plasma levels of androgens and corticosterone in anurans from the Brazilian semi-arid. Gen Comp Endocrinol 228: 9–16. 26808964 10.1016/j.ygcen.2016.01.011

[ref80] Mansour N, Lahnsteiner F, Patzner RA (2011) Collection of gametes from live axolotl, *Ambystoma mexicanum*, and standardization of in vitro fertilization. Theriogenology 75: 354–361. 20965554 10.1016/j.theriogenology.2010.09.006

[ref151] Marcec RM (2016) Development of assisted reproductive technologies for endangered North American salamanders. PhD dissertation, Mississippi State University.

[ref81] McDonough CE, Martin MW, Vance CK, Cole JA, Kouba AJ (2016) Frequency of exogenous hormone therapy impacts spermiation in male Fowler's toad (*Bufo fowleri*). *Reprod Fertil* Dev 28: 995–1003. 25585046 10.1071/RD14214

[ref82] McFadden M, Hobbs R, Marantelli G, Harlow P, Banks C, Hunter D (2013) Captive management and breeding of the critically endangered southern corroboree frog *(Pseudophryne corroboree)* (Moore 1953) at Taronga and Melbourne Zoos. Amphib Reptile Conserv 5: 70–87.

[ref83] McFadden M, Hunter D, Evans M, Scheele B, Pietsch R, Harlow P (2016) Re-introduction of the northern corroboree frog in the Northern Brindabella Mountains, New South Wales, Australia. Global Re-introduction Perspectives: 2016 Case-studies from around the globe. Gland, Switzerland: IUCN/SSC Re-introduction Specialist Group and Abu Dhabi, UAE: Environment Agency, pp. 35–39.

[ref84] McFadden MS, Gilbert D, Bradfield K, Evans M, Marantelli G, Byrne P (2018) Chapter 11 The role of ex-situ amphibian conservation in Australia. In Status of Conservation and Decline of Amphibians. CSIRO Publishing, Australia, New Zealand, and Pacific Islands, pp. 125–140.

[ref85] McWilliams DA (2008) Nutrition recommendations for some captive amphibian species (Anura and Caudata) Can Assoc Zoo Aquariums Nutr Advis Res Group, 34. http://www.amphibianark.org/wp-content/uploads/2018/07/Amphibian-nutrition-report-CAZA-2008.pdf.

[ref86] Mendonça M, Licht P, Ryan MJ, Barnes R (1985) Changes in hormone levels in relation to breeding behavior in male bullfrogs *(Rana catesbeiana)* at the individual and population levels. Gen Comp Endocrinol 58: 270–279. 3873380 10.1016/0016-6480(85)90343-0

[ref87] Miyazaki T, Tokumoto T (2013) A novel method for induction of pairing in Xenopus by addition of steroids into the water. Zoolog Sci 30: 565–569. 23829209 10.2108/zsj.30.565

[ref88] Michael S, Buckley C, Toro E, Estrada A, Vincent S (2004) Induced ovulation and egg deposition in the direct developing anuran *Eleutherodactylus coqui*. Reprod Biol Endocrinol 2: 6. 14748925 10.1186/1477-7827-2-6 PMC340388

[ref89] Millar RP, Lu Z-L, Pawson AJ, Flanagan CA, Morgan K, Maudsley SR (2004) Gonadotropin-releasing hormone receptors. Endocr Rev 25: 235–275. 15082521 10.1210/er.2003-0002

[ref90] Minteer BA, Collins JP (2013) Ecological ethics in captivity: balancing values and responsibilities in zoo and aquarium research under rapid global change. ILAR J 54: 41–51. 23904531 10.1093/ilar/ilt009

[ref91] Moore FL (1987) Reproductive endocrinology of amphibians. In Fundamentals of Comparative Vertebrate Endocrinology. Springer, New York, pp. 207–221.

[ref92] Moore FL, Miller LJ (1984) Stress-induced inhibition of sexual behavior: corticosterone inhibits courtship behaviors of a male amphibian *(Taricha granulosa)*. Horm Behav 18: 400–410. 6097527 10.1016/0018-506x(84)90026-6

[ref93] Moore FL, Zoeller RT (1985) Stress-induced inhibition of reproduction: evidence of suppressed secretion of LH-RH in an amphibian. Gen Comp Endocrinol 60: 252–258. 3905502 10.1016/0016-6480(85)90321-1

[ref94] Moore IT, Jessop TS (2003) Stress, reproduction, and adrenocortical modulation in amphibians and reptiles. Horm Behav 43: 39–47. 12614633 10.1016/s0018-506x(02)00038-7

[ref95] Nagel AH, Beshel M, DeChant CJ, Huskisson SM, Campbell MK, Stoops MA (2019) Non-invasive methods to measure inter-renal function in aquatic salamanders—correlating fecal corticosterone to the environmental and physiologic conditions of captive Necturus. Conserv Physiol 7: coz074. 10.1093/conphys/coz074 PMC6845813 31737273

[ref96] Narayan EJ, Hero J-M, Cockrem JF (2012a) Inverse urinary corticosterone and testosterone metabolite responses to different durations of restraint in the cane toad *(Rhinella marina)*. Gen Comp Endocrinol 179: 345–349. 23036735 10.1016/j.ygcen.2012.09.017

[ref97] Narayan EJ, Molinia FC, Cockrem JF, Hero J-M (2012b) Individual variation and repeatability in urinary corticosterone metabolite responses to capture in the cane toad *(Rhinella marina)*. Gen Comp Endocrinol 175: 284–289. 22137908 10.1016/j.ygcen.2011.11.023

[ref98] NHMRC E (2008) Part III Administration of substances, Guidelines to Promote the Wellbeing of Animals Used for Scientific Purposes: The Assessment and Alleviation of Pain and Distress in Research Animals. Australian Government National Health and Medical Research Council, pp, pp. A1–A10 https://www.nhmrc.gov.au/about-us/publications/guidelines-promote-wellbeing-animals-used-scientific-purposes#block-views-block-file-attachments-content-block-1

[ref99] O’Brien DM, Keogh JS, Silla AJ, Byrne PG (2019) Female choice for related males in wild red-backed toadlets (Pseudophryne coriacea). Behav Ecol 30: 928–937.

[ref100] O’Rourke DP, Baccanale CL, Stoskopf MK (2018) Nontraditional laboratory animal species (cephalopods, fish, amphibians, reptiles, and birds). ILAR J 59: 168–176. 30462255 10.1093/ilar/ily003

[ref101] Ogawa A, Dake J, Y-k I, Tokumoto T (2011) Induction of ovulation in Xenopus without hCG injection: the effect of adding steroids into the aquatic environment. Reprod Biol Endocrinol 9: 11. 21255406 10.1186/1477-7827-9-11 PMC3032665

[ref102] Ogielska M (2009) *Reproduction of Amphibians*. Science Publishers, Enfield, NH, USA, p. 422

[ref103] Ogilvy V, Preziosi R, Fidgett A (2012) A brighter future for frogs? The influence of carotenoids on the health, development and reproductive success of the red-eye tree frog. Anim Conserv 15: 480–488.

[ref104] Orchinik M, Licht P, Crews D (1988) Plasma steroid concentrations change in response to sexual behavior in *Bufo marinus*. Horm Behav 22: 338–350. 3139541 10.1016/0018-506x(88)90006-2

[ref105] Osborne WS (1991) The biology and management of the corroboree frog (*Pseudophryne corroboree*) in NSW. Species management report. New South Wales National Parks and Wildlife Service, Hurstville, NSW. no. 8.

[ref106] Peter RE, Lin HR, Van Der Kraak G (1988) Induced ovulation and spawning of cultured freshwater fish in China: advances in application of GnRH analogues and dopamine antagonists. Aquaculture 74: 1–10.

[ref107] Polzonetti-Magni A, Mosconi G, Carnevali O, Yamamoto K, Hanaoka Y, Kikuyama S (1998) Gonadotropins and reproductive function in the anuran amphibian, *Rana esculenta*. Biol Reprod 58: 88–93. 9472927 10.1095/biolreprod58.1.88

[ref108] Poo S, Hinkson KM, Stege E (2019) Sperm output and body condition are maintained independently of hibernation in an endangered temperate amphibian. Reprod Fertil Dev 31: 796–804. 30514432 10.1071/RD18073

[ref109] Popesku JT, Martyniuk CJ, Mennigen J, Xiong H, Zhang D, Xia X, Cossins AR, Trudeau VL (2008) The goldfish *(Carassius auratus)* as a model for neuroendocrine signaling. Mol Cell Endocrinol 293: 43–56. 18657592 10.1016/j.mce.2008.06.017

[ref110] Pramuk JB, Gagliardo R (2012) General amphibian husbandry. In Amphibian Husbandry Resource Guide, Edition 2, pp. 4–59.

[ref111] Pritchard DJ, Fa JE, Oldfield S, Harrop SR (2012) Bring the captive closer to the wild: redefining the role of *ex situ* conservation. Oryx 46: 18–23.

[ref112] Romero LM (2002) Seasonal changes in plasma glucocorticoid concentrations in free-living vertebrates. Gen Comp Endocrinol 128: 1–24. 12270784 10.1016/s0016-6480(02)00064-3

[ref113] Romero LM, Dickens MJ, Cyr NE (2009) The reactive scope model—a new model integrating homeostasis, allostasis, and stress. Horm Behav 55: 375–389. 19470371 10.1016/j.yhbeh.2008.12.009

[ref114] Roth T, Szymanski D, Keyster E (2010) Effects of age, weight, hormones, and hibernation on breeding success in boreal toads *(Bufo boreas boreas)*. Theriogenology 73: 501–511. 20004010 10.1016/j.theriogenology.2009.09.033

[ref115] Roth TL, Obringer AR (2003) Reproductive research and the worldwide amphibian extinction crisis. In WV Holt, AR Pickard, JC Rodger, DE Wildt, eds. In Reproductive Science and Integrated Conservation. Cambridge University Press, Cambridge, UK, pp. 359–374.

[ref116] Rowson AD, Obringer AR, Roth TL (2001) Non-invasive treatments of luteinizing hormone-releasing hormone for inducing spermiation in American *(Bufo americanus)* and Gulf Coast *(Bufo valliceps)* toads. Zoo Biol 20: 63–74. 11429778 10.1002/zoo.1007

[ref117] Santana FE, Swaisgood RR, Lemm JM, Fisher RN, Clark RW (2015) Chilled frogs are hot: hibernation and reproduction of the endangered mountain yellow-legged frog *Rana muscosa*. Endanger Species Res 27: 43–51.

[ref118] Silla AJ (2010) Effects of luteinizing hormone-releasing hormone and arginine-vasotocin on the sperm-release response of Günther’s Toadlet, *Pseudophryne guentheri*. Reprod Biol Endocrinol 8: 139. 21059269 10.1186/1477-7827-8-139 PMC2992061

[ref119] Silla AJ (2011) Effect of priming injections of luteinizing hormone-releasing hormone on spermiation and ovulation in Günther’s Toadlet, *Pseudophryne guentheri*. Reprod Biol Endocrinol 9: 68. 21599916 10.1186/1477-7827-9-68 PMC3141644

[ref120] Silla AJ (2013) Artificial fertilisation in a terrestrial toadlet (*Pseudophryne guentheri*): effect of medium osmolality, sperm concentration and gamete storage. Reprod Fertil Dev 25: 1134–1141. 23174151 10.1071/RD12223

[ref121] Silla AJ, Byrne PG (2019) The role of reproductive technologies in amphibian conservation breeding programs. Annu Rev Anim Biosci 7: 10.1–10.21. 10.1146/annurev-animal-020518-115056 30359086

[ref122] Silla AJ, Byrne PG (2021) Hormone-induced ovulation and artificial fertilisation in four terrestrial-breeding anurans. Reprod Fertil Dev (in press). 10.1071/RD20243 33640035

[ref123] Silla AJ, Roberts JD, Byrne PG (2020) The effect of injection and topical application of hCG and GnRH agonist to induce sperm-release in the roseate frog *Geocrinia rosea*. Conserv Physiol 8: coaa104. 10.1093/conphys/coaa104 PMC7720084 33304589

[ref124] Silla AJ, McFadden M, Byrne PG (2018) Hormone-induced spawning of the critically endangered northern corroboree frog *Pseudophryne pengilleyi*. Reprod Fertil Dev 30: 1352–1358. 29694827 10.1071/RD18011

[ref125] Silla AJ, McFadden MS, Byrne PG (2019) Hormone-induced sperm-release in the critically endangered Booroolong frog (*Litoria booroolongensis*): effects of gonadotropin-releasing hormone and human chorionic gonadotropin. Conserv Physiol 7: coy080. 10.1093/conphys/coy080 PMC6372942 30792859

[ref126] Silla AJ, Roberts JD (2012) Investigating patterns in the spermiation response of eight Australian frogs administered human chorionic gonadotropin (hCG) and luteinizing hormone-releasing hormone (LHRHa). Gen Comp Endocrinol 179: 128–136. 22909973 10.1016/j.ygcen.2012.08.009

[ref23] Smalley KN , Nace GW (1983) Vitellogenic cycles in laboratory-maintained females of the leopard frog, *Rana pipiens*. Journal of Experimental Zoology 226: 211–219. 10.1002/jez.1402260206 6602862

[ref127] Son HH, Yun WS, Cho SH (2020) Development and validation of an LC-MS/MS method for profiling 39 urinary steroids (estrogens, androgens, corticoids, and progestins). Biomed Chromatogr 34: e4723. 31656044 10.1002/bmc.4723

[ref128] Sotowska-Brochocka J (1988) The stimulatory and inhibitory role of the hypothalamus in the regulation of ovulation in grass frog, *Rana temporaria* L. Gen Comp Endocrinol 70: 83–90. 3286371 10.1016/0016-6480(88)90096-2

[ref129] Sotowska-Brochocka J, Licht P (1992) Effect of infundibular lesions on GnRH and LH release in the frog, *Rana temporaria,* during hibernation. Gen Comp Endocrinol 85: 43–54. 1563617 10.1016/0016-6480(92)90170-o

[ref130] Sotowska-Brochocka J, Martyńska L, Licht P (1994) Dopaminergic inhibition of gonadotropic release in hibernating frogs, *Rana temporaria*. Gen Comp Endocrinol 93: 192–196. 8174925 10.1006/gcen.1994.1022

[ref131] Stebbins RC (1951). Amphibians of Western North America. University of California Press; 539.

[ref132] Stearns SC (1992) The Evolution of Life Histories. Oxford University Press, Oxford, UK.

[ref133] Taylor AC (2003) Assessing the consequences of inbreeding for population fitness: past challenges and future prospects. In WV Holt, AR Pickard, JC Rodger, DE Wildt, eds,. In Reproductive Science and Integrated Conservation. Cambridge University Press, Cambridge, UK, pp. 67–81.

[ref134] Teitsma C, Lethimonier C, Tujague M, Anglade I, Saligaut D, Bailhache T, Pakdel F, Kah O, Ducouret B (1998) Identification of potential sites of cortisol actions on the reproductive axis in rainbow trout. Comp Biochem Physiol Part C Pharmacol Toxicol Endocrinol 119: 243–249. 10.1016/s0742-8413(98)00013-9 9826997

[ref135] Toledo LF, Sazima I, Haddad CFB (2011) Behavioural defences of anurans: an overview. Ethol Ecol Evol 23: 1–25.

[ref136] Trudeau VL, Schueler FW, Navarro-Martin L, Hamilton CK, Bulaeva E, Bennett A, Fletcher W, Taylor L (2013) Efficient induction of spawning of northern leopard frogs *(Lithobates pipiens)* during and outside the natural breeding season. Reprod Biol Endocrinol 11: 14. 23442383 10.1186/1477-7827-11-14 PMC3598769

[ref137] Trudeau VL, Somoza GM (2020) Multimodal hypothalamo-hypophysial communication in the vertebrates. Gen Comp Endocrinol 293: 113475. 32240708 10.1016/j.ygcen.2020.113475

[ref138] Trudeau VL, Somoza GM, Natale GS, Pauli B, Wignall J, Jackman P, Doe K, Schueler FW (2010) Hormonal induction of spawning in 4 species of frogs by coinjection with a gonadotropin-releasing hormone agonist and a dopamine antagonist. Reprod Biol Endocrinol 8: 1–9. 20398399 10.1186/1477-7827-8-36 PMC2873446

[ref139] Turner PV, Brabb T, Pekow C, Vasbinder MA (2011) Administration of substances to laboratory animals: routes of administration and factors to consider. J Am Assoc Lab Anim Sci 50: 600–613. 22330705 PMC3189662

[ref140] Uteshev V, Kaurova S, Shishova N, Stolyarov S, Browne R, Gakhova E (2015) In vitro fertilization with hormonally induced sperm and eggs from sharp-ribbed newts *Pleurodeles waltl*. Russ J Herpetol 22.

[ref141] Vu M, Trudeau VL (2016) Neuroendocrine control of spawning in amphibians and its practical applications. Gen Comp Endocrinol 234: 28–39. 27013378 10.1016/j.ygcen.2016.03.024

[ref142] Vu M, Weiler B, Trudeau VL (2017) Time- and dose-related effects of a gonadotropin-releasing hormone agonist and dopamine antagonist on reproduction in the northern leopard frog *(Lithobates pipiens)*. Gen Comp Endocrinol 254: 86–96. 28964731 10.1016/j.ygcen.2017.09.023

[ref143] Wang L, Ahn RS, Park J-Y, Seong JY, Kwon HB (2003) Differential G protein coupling preference of mammalian and nonmammalian gonadotropin-releasing hormone receptors. Mol Cell Endocrinol 205: 89–98. 12890570 10.1016/s0303-7207(03)00204-1

[ref144] Wang L, Bogerd J, Choi HS, Seong JY, Soh JM, Chun SY, Blomenröhr M, Troskie BE, Millar RP, Wen HY (2001) Three distinct types of GnRH receptor characterized in the bullfrog. Proc Natl Acad Sci 98: 361–366. 11120886 10.1073/pnas.011508498 PMC14595

[ref145] Wells KD (2007) Chapter 10: The Natural History of Amphibian Reproduction. In KD Wells, ed. In The Ecology and Behavior of Amphibians. University of Chicago Press, Chicago

[ref146] Whitaker BR (2001) Reproduction. In BR Whitaker, ed,. In Amphibian Medicine and Captive Husbandry. Wright KM. Krieger Publishing Company, pp. 285–299.

[ref147] Whittier JM, Crews D (1987) Seasonal reproduction: patterns and control. *Hormones and Reproduction in Fishes, Amphibians, and Reptiles*. Springer, Boston, MA, pp. 385–409.

[ref148] Woodley SK, Lacy EL (2010) An acute stressor alters steroid hormone levels and activity but not sexual behavior in male and female Ocoee salamanders *(Desmognathus ocoee)*. Horm Behav 58: 427–432. 20580723 10.1016/j.yhbeh.2010.05.011

[ref149] Wren S, Angulo A, Meredith H, Kielgast J, Dos Santos M, Bishop P (2015) Amphibian Conservation Action Plan. IUCN SSC Amphibian Specialist Group, https://www.iucn-amphibians.org/resources/acap/

[ref150] Wright KM (2001) Restraint techniques and euthanasia. In BR Whitaker, ed. In Amphibian Medicine and Captive Husbandry. Wright KM. Krieger Publishing Company, pp. 111–122.

[ref152] Yamanouchi H, Ishii S (1990) Positive cooperative action of follicle-stimulating hormone on binding of luteinizing hormone to testicular receptors from the bullfrog (*Rana catesbeiana*). Gen Comp Endocrinol 78: 231–241. 2113018 10.1016/0016-6480(90)90010-j

[ref153] Yao M, Hu F, Denver RJ (2008) Distribution and corticosteroid regulation of glucocorticoid receptor in the brain of *Xenopus laevis*. J Comp Neurol 508: 967–982. 18399546 10.1002/cne.21716

